# NMR-Verified Dearomatization of 5,7-Substituted Pyrazolo[1,5-a]pyrimidines

**DOI:** 10.3390/molecules28186584

**Published:** 2023-09-12

**Authors:** Daria Novikova, Ammar Al Mustafa, Tatyana Grigoreva, Svetlana Vorona, Stanislav Selivanov, Vyacheslav Tribulovich

**Affiliations:** 1 Laboratory of Molecular Pharmacology, St. Petersburg State Institute of Technology, St. Petersburg 190013, Russia; ammar.almus@hotmail.com (A.A.M.); rozentatiana@gmail.com (T.G.); s.vorona@bk.ru (S.V.); 2 Laboratory of Biomolecular NMR, St. Petersburg State University, St. Petersburg 199034, Russia; nmr.group.spbu@gmail.com; 3 Department of Organic Chemistry, St. Petersburg State Institute of Technology, St. Petersburg 190013, Russia

**Keywords:** pyrazolo[1,5-a]pyrimidine, dearomatization, NOESY, proton–proton vicinal constants, conformational analysis, long-range interproton distance, t_1_ noise, t_2_ noise

## Abstract

Tetrahydropyrazolo[1,5-a]pyrimidine (THPP) is an attractive scaffold for designing biologically active compounds. The most obvious way to obtain such compounds is to reduce pyrazolopyrimidines with complex hydrides, because the pyrimidine ring is reduced in the preference over the pyrazole ring. The presence of substituents at positions five and seven of pyrazolo[1,5-a]pyrimidines complicates the set of reaction products but makes it more attractive for medicinal chemistry because four possible stereoisomers can be formed during reduction. However, the formation of only *syn*-isomers has been described in the literature. This article is the first report on the formation of *anti*-configured isomers along with *syn*-isomers in the reduction of model 5,7-dimethylpyrazolo[1,5-a]pyrimidine, which was confirmed by NMR. The bicyclic core in the *syn*-configuration was shown to be conformationally stable, which was used to estimate the long-range interproton distances using NOESY data. At the same time, long-range dipole–dipole interactions corresponding to a distance between protons of more than 6 Å were first registered and quantified. In turn, the bicyclic core in the *trans*-configuration represents a conformationally labile system. For these structures, an analysis of conformations observed in solutions was carried out. Our results indicate the significant potential of *trans*-configured tetrahydropyrazolo[1,5-a]pyrimidines for the development of active small molecules. While possessing structural lability due to the low energy of the conformational transition, they have the ability to adjust to the active site of the desired target.

## 1. Introduction

Almost all applications of NMR spectroscopy in the structural and conformational analysis of small molecules in solution involve obtaining information based on the Nuclear Overhauser Effect (NOE) [1,2,3] and/or using the Karplus dependence of the vicinal constants ^3^*J*_H–H_ on the dihedral angle θ [4,5]. One of the key moments in the formation and development of various NMR approaches to the study of the spatial structure and intramolecular mobility was the emergence of COSY and NOESY correlation methods, which significantly expanded the possibilities of NMR spectroscopy [6], especially in the study of large biomolecular systems with complex overlapping spectrum regions but with a higher sensitivity to the detection of NOEs compared with small molecules [7,8].

Structural NMR studies are of particular importance in drug development. Although X-ray crystallography plays critical roles in determining the structures of protein complexes with small molecule ligands, NMR spectroscopy is able to provide information on structural features, tautomerism, and ligand–receptor interactions. One of the basic concepts in structure-based drug discovery is the scaffold, which underlies the majority of biologically active small molecules. This structural element allows the introduction and specific orientation of various substituents, allowing the final molecule to be adjusted to the binding cavity of a specific target. Compounds containing the same scaffold represent structures of a chemotype and can often be synthesized in a similar way according to the concept of the molecular template in combinatorial chemistry [9]. Using a suitable scaffold, one can analyze substitution patterns and, as a result, construct structure–activity relationships [10]. The scaffold approach shows several advantages; in particular, its outcomes are both simple to interpret and medicinal chemistry oriented [11].

Pyrazolo[1,5-a]pyrimidine moiety is widely used for the design of small molecules with diverse biological activity and can be found in the structure of such drugs as indiplon, zaleplon, presatovir, dinaciclib, and anagliptin [12]. One of the features of this motif from the point of view of chemical synthesis is its nontrivial behavior in reduction reactions. When such pyrazolopyrimidines are reacted with NaBH_4_, a pyrimidine ring is reduced in the preference over the pyrazole ring and amide and ester groups if they are present in the structure [13]. A particularly interesting situation arises when considering 5,7-substituted pyrazolo[1,5-a]pyrimidines obtained by the interaction of various β-dicarbonyl compounds and their synthetic analogues with 5-aminopyrazoles, because four possible stereoisomers can be formed during reduction upon indicated conditions. Due to significant differences in the spatial orientation, they will ultimately possess different levels of the target activity. However, the formation of only *syn*-isomers, (5*S*,7*R*) and (5*R*,7*S*) enantiomers, has been described to date in the literature [14,15,16].

The presence of aromatic fragments in biologically active molecules provides an increase in the dimensionality due to an increase in the content of sp^3^ hybridized carbon atoms through dearomatization/reduction of cyclic elements of the structure. It is assumed that more highly complex molecules have the capacity to access greater chemical space [17], which results in a greater potential to identify compounds that better complement the spatial subtleties of target proteins [18,19]. The fine spatial orientation of substituents is especially important in the case of the conformational rearrangement of a protein induced by small molecules [20,21].

**Figure 1:**
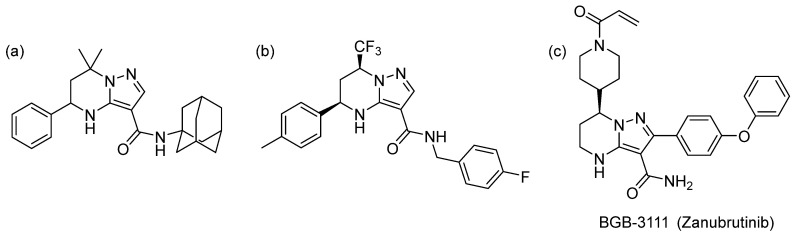
Structures of the most active THPP representatives of CaSR antagonists (**a**), antituberculous agents (**b**), and Bruton’s tyrosine kinase inhibitors (**c**).

A tetrahydropyrazolo[1,5-a]pyrimidine (THPP) scaffold is not as common as the privileged pyrazolo[1,5-a]pyrimidine moiety; however, calcium-sensing receptor (CaSR) antagonists (Figure 1a) [14], oral antituberculous agents (Figure 1b) [15], and Bruton’s tyrosine kinase inhibitors (Figure 1c) [22] can be found among its derivatives. The possibility of using tetrahydropyrazolo[1,5-a]pyrimidine as an adenine mimetic for binding to the ATP-binding sites of proteins is of particular interest [23,24]. Reduction of the aromatic pyrimidine ring of pyrazolo[1,5-a]pyrimidines to tetrahydropyrazolo[1,5-a]pyrimidines is a clear example of increasing complexity [25] and dimensionality of the structure, which is in line with the “escape from flatland” trends in drug design of recent decades [17].

In this article, dearomatization of the pyrimidine ring of pyrazolo[1,5-a]pyrimidine moiety via reduction is considered through the example of model ethyl 5,7-dimethylpyrazolo[1,5-a]pyrimidine-3-carboxylate. It is shown that such dearomatization results in two pairs of geometric isomers, which have significantly different conformational lability, as predicted by calculations and confirmed by NMR experiments. The resulting set of isomers was shown to be a good model object for demonstrating the advances of considering vicinal spin–spin coupling constants and concurrent NOE analysis for structural and conformational NMR studies of bioactive small molecules.

## 2. Results

### 2.1. Chemistry

**Scheme 1:**
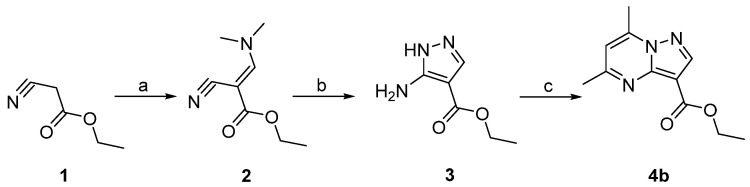
Synthesis of ethyl 5,7-dimethylpyrazolo[1,5-a]pyrimidine-3-carboxylate. Reagents and conditions: (a) (MeO)_2_CHNMe_2_, 70 °C, 6 h, 90%; (b) NH_2_NH_2_∙H_2_O, EtOH/H_2_O, 90 °C, 4 h, 82%; (c) CH_3_COCH_2_COCH_3_, AcOH/EtOH, 100 °C, 6 h, 80%.

Ethyl 5,7-dimethylpyrazolo[1,5-a]pyrimidine-3-carboxylate was chosen as a model 5,7-substituted pyrazolo[1,5-a]pyrimidine substrate because it is one of the simplest disubstituted derivatives and it can be obtained from available reagents. Due to the widespread use of pyrazolopyrimidines in the design of bioactive molecules, a large number of synthetic strategies have been developed to obtain them. We prepared the desired pyrazolopyrimidine according to the methodology for constructing the pyrazolo[1,5-a]pyrimidine core described in [26] in three steps from ethyl 2-cyanoacetate (**1**) without isolating the intermediate ethyl 2-cyano-3-(dimethylamino)acrylate (**2**) (Scheme 1).

**Scheme 2:**
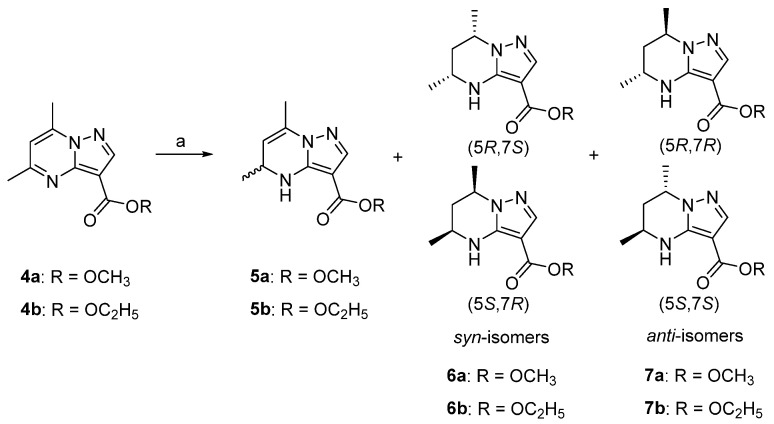
Dearomatization of 5,7-dimethylpyrazolo[1,5-a]pyrimidine-3-carboxylate. Reagents and conditions: (a) NaBH_4_, ROH, RONa, R = CH_3_, and C_2_H_5_.

The obtained pyrazolopyrimidine (**4**) was introduced into the reduction reaction with sodium borohydride in methanol catalyzed by sodium methoxide. This reduction protocol was chosen as the most accessible and studied method for the reduction of the carbonyl group. However, it does not lead to a reduced carbonyl group in pyrazolo[1,5-a]pyrimidines. The result of the reaction was a complex mixture of products, which is described in Scheme 2.

Our efforts were aimed at isolating a previously undescribed reaction product; namely, a substrate in the *anti*-configuration. The use of methyl 5,7-dimethylpyrazolo[1,5-a]pyrimidine-3-carboxylate (**4a**) as the initial reagent led to an inseparable mixture of methyl esters of the reduced substrate. However, the reduction of ethyl 5,7-dimethylpyrazolo[1,5-a]pyrimidine-3-carboxylate (**4b**) in ethanol allowed us to first isolate and characterize the reduction product of 5,7-substituted pyrazolo[1,5-a]pyrimidine in the *anti*-configuration (**7b**). The total yield of all reduction products was very high in the case of the reduction in methanol (up to 95%), and slightly lower for the reduction protocol in ethanol (85%). It should be noted that in all reactions, the product of the partial reduction of the pyrimidine ring, dihydropropyrazolopyrimidine (**5a**,**b**), the structure of which, according to NMR spectroscopy data, confirms the previously proposed two-stage mechanism [13], was detected.

The ratio of tetrahydropyrazolopyrimidine products in the *syn*- and *anti*-configurations during reduction with NaBH_4_ was 7:1 in favor of the *syn*-isomer. Changing the reaction conditions—in particular, the use of tetrabutylammonium borohydride in an aprotic solvent, such as chloroform—led to an increase in the proportion of the *anti*-isomer to a ratio of 1:1.

The structure of the pyrazolopyrimidine reduction products was established based on NMR experiments considering MS data. For all the products, the disappearance of the aromatic signal H^6^ at 7.08 ppm and the appearance of the nitrogen proton NH^4^ were noted (Figures S2, S4, S6 and S12). For dihydropropyrazolopyrimidine **5b,** the letter signal has a coupling constant of 2.0 Hz, unambiguously pointing to the persistence of the double bond between C^6^ and C^7^. The stereochemical assignment of the isolated tetrahydropyrazolopyrimidines **6b** and **7b** was made based on NOESY data. The presence of the cross-peak between protons H^5^ and H^7^ for **6b** (Figure S11) indicates their spatial proximity, which is possible only in the case of the *cis*-configuration of methyl substituents at positions five and seven, while no such signal is observed for **7b** (Figure S17).

### 2.2. Computational Studies

Computer simulation of the studied compounds in order to calculate the expected values of dihedral angles and interproton distances, which are compared with their experimental data, is the key to the effective use of vicinal spin–spin coupling constants and NOE. CASE (Computer Assisted Structure Elucidation) is currently actively used to study the structure of natural compounds [27,28]. This approach turns out to be useful in the analysis of complex proton systems within quantitative NMR (qNMR) [29,30].

We performed conformational energy calculations for all four stereoisomers **6b** and **7b**, which are reduction products of the pyrimidine ring of ethyl 5,7-dimethylpyrazolo[1,5-a]pyrimidine-3-carboxylate (**4b**). As expected, we did not record any differences within the enantiomeric pairs both in terms of the potential energy and the geometric parameters of the generated conformers. Therefore, hereinafter, all characteristics for *syn*-isomers will be given using the example of ethyl (5*R*, 7*S*)-5,7-dimethyl-4,5,6,7-tetrahydropyrazolo[1,5-a]pyrimidine-3-carboxylate (**6b**) indicated as SYN, while *anti*-isomers will be exemplified by ethyl (5*S*, 7*S*)-5,7-dimethyl-4,5,6,7-tetrahydropyrazolo[1,5-a]pyrimidine-3-carboxylate (**7b**) indicated as ANTI.

**Figure 2:**
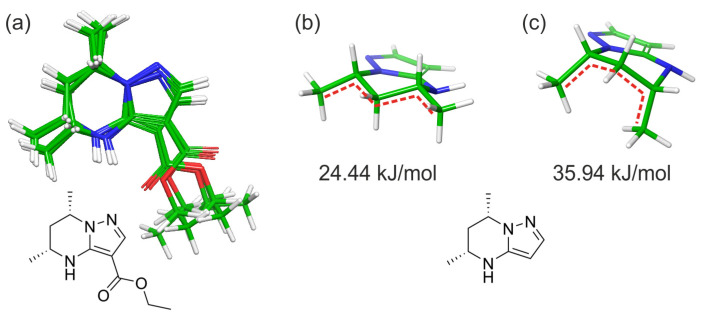
Superposition of main SYN conformations (**a**) and two possible conformations of the pyrazolopyrimidine core (**b**,**c**). Legend: carbon, hydrogen, nitrogen, and oxygen atoms are given in green, white, blue, and red, respectively.

According to our calculations, the conformational diversity of SYN is determined by the rotation of the ester group around the C–C bond with the pyrazole ring (Figure 2a). The energy spread for the generated conformations is 6.7 kJ/mol. The geometry of the pyrimidine core remains unchanged. For the bicyclic fragment of the SYN isomer, only two conformations with an energy difference of 11.5 kJ/mol are possible (Figure 2b,c), which reduces the likelihood of the existence of a SYN conformation with an axial arrangement of methyl groups (Figure 2c).

**Figure 3:**
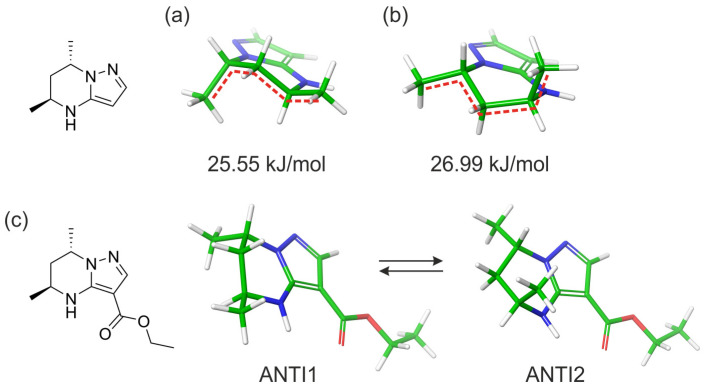
Two possible conformations of the pyrazolopyrimidine core for the *anti*-configuration of substituents: 5-CH_3_ at equatorial position, 7-CH_3_ at axial position (**a**); 5-CH_3_ at axial position, 7-CH_3_ at equatorial position (**b**); and the transition between the most energetically favorable ANTI conformations (**c**). Legend: carbon, hydrogen, nitrogen, and oxygen atoms are given in green, white, blue and red, respectively.

In the case of ANTI, two main configurations of the pyrimidine ring are observed: the configuration with a C^6^ atom located above the plane of the molecule, in which a methyl group at position seven occupies the axial position (Figure 3a), and the configuration with C^6^ atom located under the plane of the molecule, in which a methyl group at position five is so far in the axial position (Figure 3b). Under the fixed orientation of the ester group in the pyrazole ring, the difference in energies between such conformations is about 1.5 kJ/mol, which suggests a rather facile interconformational transition for the ANTI molecule (Figure 3c).

### 2.3. NMR Studies

#### 2.3.1. Analysis of Vicinal Constants to Study the Spatial Structure and Intramolecular Flexibility of Reaction Products

Vicinal ^1^H–^1^H coupling constants comprise the vast majority of coupling data that have been reported. Since the theoretical dependence of vicinal coupling constants on the dihedral angle of the coupled protons, known as the Karplus equation, was proposed [31], it has been increasingly used for establishing the configuration and conformational features of organic compounds in solutions.

The main problems in the practical application of this spectral parameter are associated with additional contributions to the ^3^*J*_H–H_ value from other factors, such as the electronegativity of substituents in α- and β-positions of the ethane fragment, the influence of bond angles, and the C–C bond length. In this regard, the original Karplus equation has been modified, extended, generalized, and reparametrized to include a large number of different nuclear pairs and more finely tuned dependences on a host of molecular properties in addition to the dihedral angle [32]. The use of the most modern modifications of the Karplus equation provides a sufficiently accurate prediction of the values of vicinal constants within 0.5–0.8 Hz in comparison with the experimental data [33,34]. The most correct estimates are the relative changes in vicinal constants for similar molecules, in which only dihedral angles differ, while the influence of other factors is leveled [35].

The experimental values of the vicinal constants in the –C^5^H–C^6^H_2_–C^7^H– fragment were determined directly from the analysis of the multiplet structure of the corresponding signals, which were then compared with the calculated values of the vicinal constants. In the case of the CIS molecule, presumably, the same large values of this constant (11.1 Hz) between the H^6^_α_ proton and protons H^5^ and H^7^ indicate their *trans*-diaxial arrangement in the studied molecule, while the small unequal values of the constants of protons H^5^ and H^7^ with the H^6^_β_ proton (2.9, 4.2 Hz, respectively) indicate a slight asymmetry of the considered fragment with respect to the H^6^_α_–C^6^–H^6^_β_ plane. The experimental values turned out to be in good agreement with the calculated values for the proposed structure (Table 1).

In the case of the ANTI molecule, the experimental values of the vicinal constants between protons H^6^_α_ and H^5^, as well as between protons H^6^_β_ and H^7^ (4.2 and 5.3 Hz, respectively), were close to the calculated values obtained for both ANTI1 and ANTI2 conformers. At the same time, the vicinal constant of the H^6^_α_ signal with the H^7^ proton, equal to 4.8 Hz, turns out to be greater than the calculated value for ANTI1 and significantly less than its calculated value for ANTI2. The opposite picture is observed for the vicinal constant of the H^6^_β_ signal with the H^5^ proton (Table 2). This suggests that these values are averaged over time, which was subsequently used to estimate the ratio of conformers in solution.

#### 2.3.2. Quantitative Evaluation of the Ratio of ANTI Conformers Based on Vicinal Constant Values

**Figure 4:**
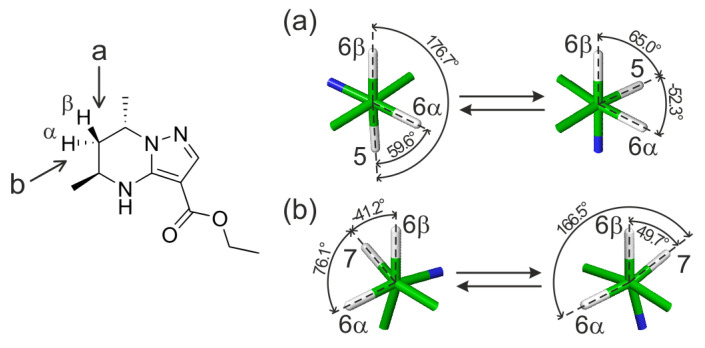
Newman projections along C^6^–C^5^ (**a**) and C^6^–C^7^ (**b**) bonds for ANTI1 and ANTI2 conformers. The direction of view is marked with arrows next to letters (a, b). Legend: carbon, hydrogen, and nitrogen atoms are given in green, white, and blue, respectively.

In the ANTI molecule, the H^6^_α_ proton forms an AB-type spin system with the geminal H^6^_β_ proton and is represented by a doublet of triplets, each component of which is additionally split into two components due to the long-range scalar interaction with the H^4^ proton with a constant of 1.1 Hz. This constant turns out to be almost two times smaller than the corresponding constant in the case of the SYN molecule, in which the H^6^_β_ proton occupies a pseudoequatorial position and forms a planar system of W-type covalent bonds [4] with the H^4^ proton. This indicates the existence of a fast dynamic equilibrium between the ANTI1 and ANTI2 conformers, and the relatively small value of the ^4^*J*_H4–H6α_ constant is a consequence of time averaging. This conformational exchange is a process of simultaneous pseudo rotation around C^6^–C^5^ and C^6^–C^7^ bonds and can be represented using Newman projections (Figure 4).

**Figure 5:**
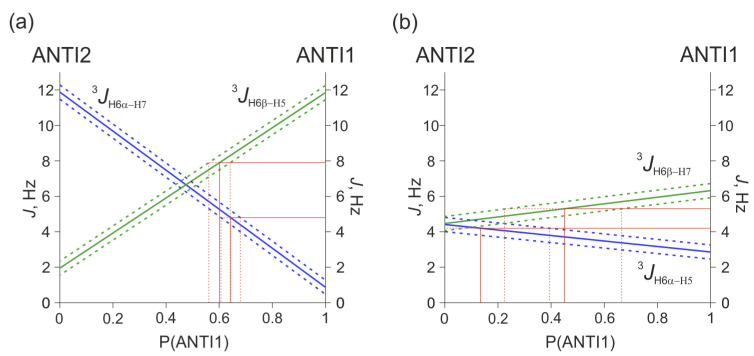
Calculated dependences of vicinal constants on the ANTI1 conformer population under fast two-position exchange: for the pair of constants ^3^*J*_H6α–H7_ and ^3^*J*_H6β–H5_ (**a**) and ^3^*J*_H6α–H5_ and ^3^*J*_H6β–H7_ (**b**). Blue and green full lines correspond to calculated dependences of corresponding constants; blue and green dashed lines indicate their errors. Red full lines are used to determine the conformer population from experimental values of corresponding constants; red dashed lines indicate errors of this value.

To quantify the ratio of conformers in DMSO-*d*_6_ solution, ^3^*J*_H6α–H7_ and ^3^*J*_H6β–H5_ were selected from the four vicinal constants in the –C^5^H–C^6^H_2_–C^7^H– fragment as the most sensitive to the equilibrium position. Based on the calculated values, graphs of the linear dependence on the population of the ANTI1 conformer—P(ANTI1) were constructed under the condition P(ANTI1) + P(ANTI2) = 1 (Figure 5a). Using the constructed graphs, for each experimental value of the selected constants (4.8 and 7.9 Hz), the corresponding values of the ANTI1 conformer population were determined (0.64 and 0.60, respectively). The calculated dependences for the constants ^3^*J*_H6α–H5_ and ^3^*J*_H6β–H7_, which change their values only within 1.5 Hz, cannot be used for accurate quantitative estimates (Figure 5b). Thus, the analysis of the vicinal constants allowed us to evaluate the ratio of ANTI1 and ANTI2 conformers as 62:38.

#### 2.3.3. Influence of Noise Bands on the Accuracy of Weak Cross-Peak Integration in NOESY Spectra

NOE spectroscopy provides information about the spatial proximity of protons, which can be used to determine the relative configuration and conformation of small and large organic molecules. NOE can be defined as a change in the signal intensity of a spin due to magnetization transfer through cross relaxation in a dipolar coupled system. Small molecules with rapid diffusion motions give rise to positive NOEs, and they can show up to fifty percent of cross-peak increase. Larger molecules show a negative NOE with a maximum signal decrease of approximately 100%. This determines advances for using NOESY data when analyzing macromolecular systems.

There are two standard approaches to analyzing NOE data: initial slope approximation and relaxation matrix analysis, although the former is more commonly used for small molecules. It requires only the values of cross-peak volume integrals at different short mixing times normalized to the corresponding diagonal integrals (S_ii_ or S_jj_) as input; no other experimental information is needed. So, the highest possible accuracy in determining cross-peak volume integrals is required when studying small molecules.

However, there are some problems related to the registration and processing of extremely small cross-peaks in NOESY spectra corresponding to long-range distances. These are an insufficient correction of the base plane in the phase-sensitive NOESY spectra, an influence of so-called “t_1_ noise” and “t_2_ noise” on the volume integral accuracy, and also overlapping of proton signals and cross-peaks. The origin of t_1_ noise and ways to deal with it are described much better [36] than the t_2_ noise phenomenon. It is assumed that the latter may be associated with the receiver overload and/or with a slight mismatch between two channels of the quadrature detection system, as well as with insufficiently accurate phase correction of the 2D spectrum.

**Figure 6:**
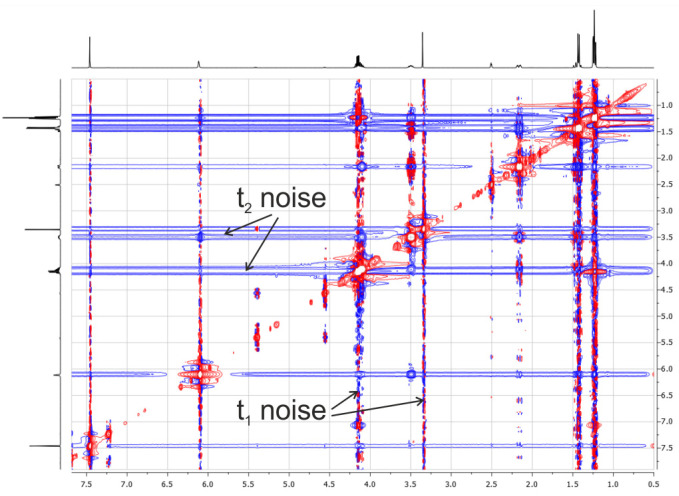
Intensified NOESY spectrum of SYN. Legend: negative and positive NOE signals are indicated in blue and red, respectively.

Long-term accumulation of the spectrum for 12 h allowed us to detect weak cross-peaks corresponding to long-range distances in the SYN molecule. However, there are t_1_ and t_2_ noise as vertical and horizontal bands at the frequencies of the NMR signals in the spectrum at high gain (Figure 6). The values of the volume integrals of the cross-peaks, which are on the t_2_ noise band, turn out to be overestimated, which leads to an underestimation of interproton distances.

To eliminate this distortion, we performed an additional integration of the t_2_ band on one (or two) sides of the measured cross-peak, and this value was subtracted from the value of the integrated intensity of the measured cross-peak. In this case, a rectangular rather than an oval integration contour was used, which gave more accurate results if its dimensions were the same for both the cross-peak and t_2_ noise. The additional integration of t_2_ noise on both sides of the cross-peak and at the same distance from which it was carried out. We emphasize the need to manually consider noise bands because cleaning the spectrum with automatic base plane alignment (Whittaker smoother algorithm) [37] and/or t_1_ noise reduction in the MestReNova program inevitably leads to the loss of a significant amount of quantitative information. This is unacceptable in cases where only long-range NOEs allow one to uniquely solve a structural or conformational problem [38].

**Figure 7:**
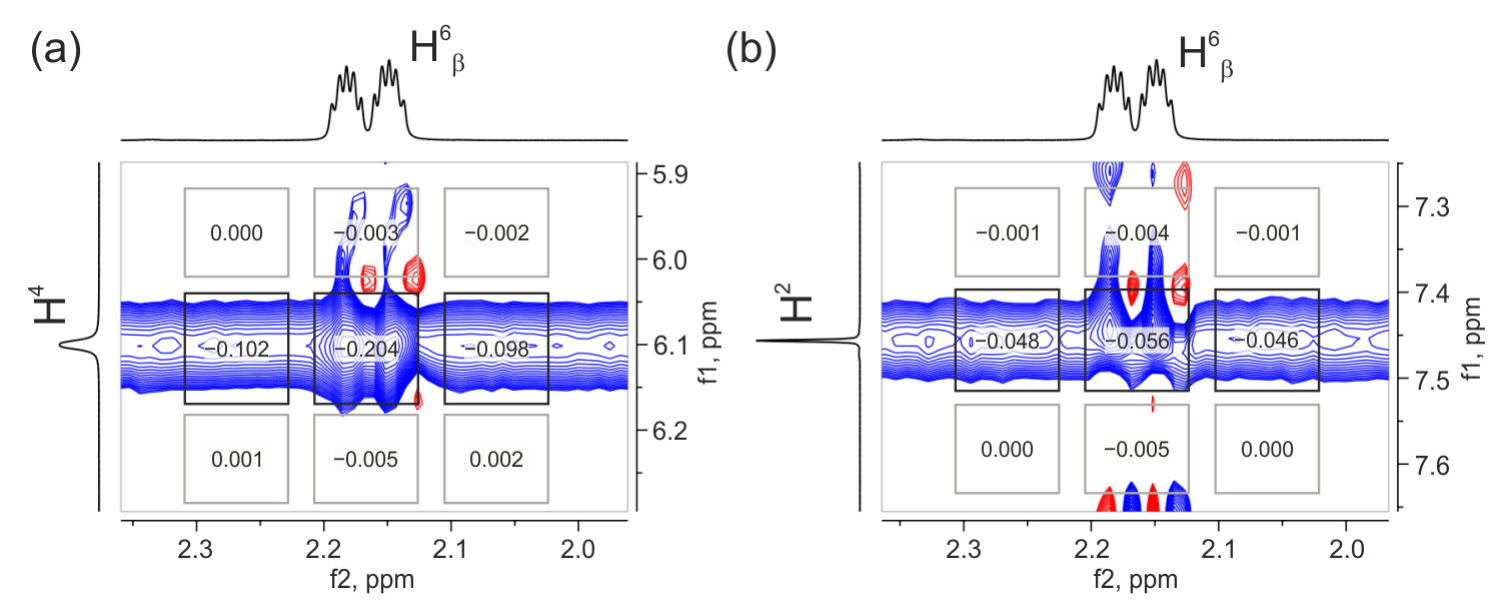
Volume integration of H^4^/H^6^_β_ (**a**) and H^2^/H^6^_β_ cross-peaks (**b**) in the NOESY spectrum of SYN. Legend: negative and positive NOE signals are indicated in blue and red, respectively.

When measuring the integral intensities of the weakest cross-peaks, the signal–noise relation was also evaluated on a region free from t_1_ and t_2_ noise bands in the vicinity of the measured cross-peak. If we look at two weak NOE signals corresponding to two long-range interproton distances (Figure 7), then the average value of the t_2_ noise contribution to the H^4^/H^6^_β_ cross-peak is almost half of the measured value (4.25 Å), and for the H^2^/H^6^_β_ cross-peak, this value reaches 84% (6.54 Å). This leads to an overestimation of the value of the integrated intensity of the weaker cross-peak of more than six times, which corresponds to a reduction in the measured distance by 26% of the measured value. The similar effect of t_2_ noise on a shorter distance is only 11% of the measured value. Thus, the error in measuring long distances depends very much on the influence of t_2_ noise, which can be significantly reduced by applying the described procedure.

At the same time, the contribution of t_1_ noise, which has a different nature and is not uniform along the vertical axis of the signal in the NOESY spectrum, to the cross-peaks above and below the measured cross-peak, averages about −0.004 and turns out to be more than 10 times less the contribution of t_2_ noise. However, it slightly exceeds the integral intensities in the signal-free rectangular regions of the integration. Consequently, the possible contribution of t_1_ noise to the cross-peaks will consist of the same increase in their intensity.

#### 2.3.4. Estimation of Interproton Distances Based on NOESY Data

The ability of NOE to detect long-range dipole–dipole interactions for small and large molecules differs. In large molecules, one can detect long-range distances up to 12 Å [39]. However, in small molecules, a proton–proton distance between 4 and 5 Å is generally accepted as the upper limit for the occurrence of measurable NOE [40]. In recent years, the understanding has been reached that for small molecules, a high accuracy of quantitative estimates of cross-relaxation rates and measurements of the corresponding distances between protons can be obtained based on the relationship σ_ij_~r_ij_^−6^, which led to the emergence of the special term exact NOE (eNOE) [41,42]. Such an analysis of the NOE data implies the estimation of distances between atoms with an accuracy of about 5%. High accuracy allows one to detect minor conformers under conditions of fast exchange [43,44] and to prove the rigidity of the structure of the studied molecule in solution [45,46].

An independent proof of the spatial structure of SYN is provided by our measurements of interproton distances and their comparison with the corresponding calculated values. The procedure for processing and integrating cross-peaks described above was also used to quantify shorter (<4.0 Å) interproton distances. It is obvious that the contributions of t_1_ and t_2_ noise to the volume integrals of such cross-peaks cannot introduce significant distortions into the distance values, as in the case of weak cross-peaks, but their elimination allows one to reveal other contributions, such as the effects of spin diffusion [47], diffusion anisotropy [48], or intramolecular mobility [49,50].

**Figure 8:**
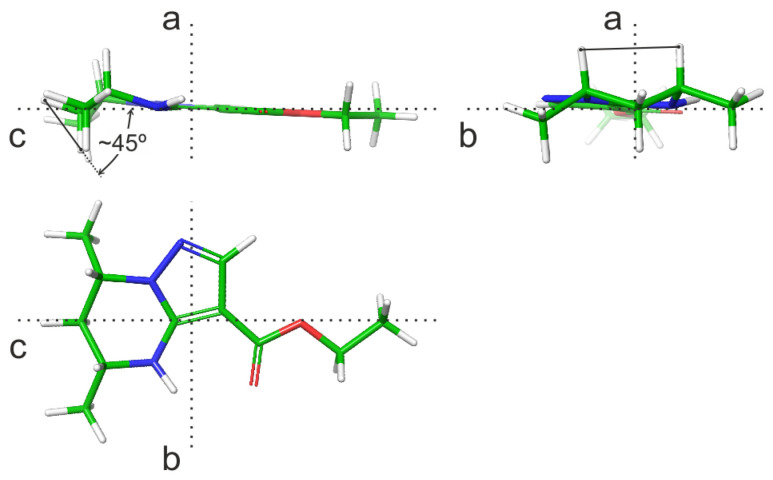
Projections of the SYN molecule along its principal axes (a–c) and determination of polar angles for radius vectors of interproton distances. Legend: carbon, hydrogen, nitrogen, and oxygen atoms are given in green, white, blue and red, respectively.

It should be noted that the anisotropy of diffusion motion also affects the accuracy of determining interproton distances based on NOE. For highly anisotropic molecules, the error in estimating interproton distances can reach 26%, and then a correction for the diffusion anisotropy is necessary. It is associated with the problem of choosing a reference distance. For our study, we used two different reference distances: r(H^5^–H^7^) = 2.69 Å and r(H^6^_α_–H^6^_β_) = 1.77 Å. These distances not only differ from each other, but they also have different spatial orientations relative to the main axis of the studied molecule. The radius vector of the first of them is located practically orthogonally to the main axis, so that the polar angle is almost 90° (Figure 8). For the second reference distance, the polar angle β is about 45°. Because, according to the calculations, the diffusion anisotropy parameter of the SYN molecule is about 4.4 (A = Dǁ/D_┴_), the possible anisotropic contribution when using the first of the standards cannot increase the cross-peak intensity by more than two times, even if the radius vector of the corresponding measured distance is parallel to the principal axis (β_ij_ = 0°).

**Figure 9:**
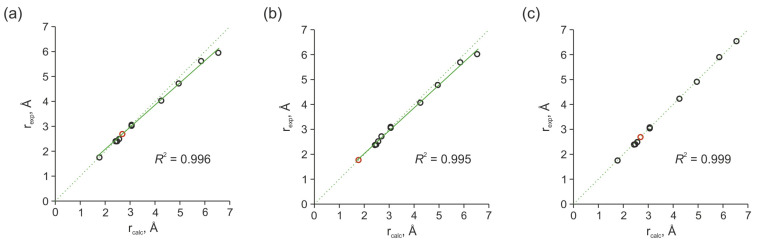
Correlation of the calculated and experimental distances (indicated as curcles) in the case of using r(H^5^–H^7^) (**a**,**c**) and r(H^6^_α_–H^6^_β_) (**b**) as reference distances (marked in red). Green full lines represent regression lines; green dashed lines represent *x* = *y* function.

In the SYN molecule, almost all polar angles for the shortest distances do not exceed 45°, while for four long distances, they are about 25–30°. Therefore, when using r(H^5^–H^7^) as the reference distance, all experimental long distances turn out to be shorter than the calculated values, while shorter distances correlate much better with the calculated values (Figure 9a). The maximum discrepancy between the data is observed for the distance r(H^2^–H^6^_β_), for which the polar angle is 20°. However, if r(H^6^_α_–H^6^_β_) is used as the reference distance, the polar angle of which is in the middle of the range, the distance correlation is closer to *x* = *y*, although the statistics slightly worsen (Figure 9b). In this case, the remaining pairs of protons have only a slight difference in polar angles in the region of the strongest change in the anisotropic contribution of the polar angle β. However, if we introduce a correction for diffusion anisotropy when using r(H^5^–H^7^) as the reference distance, i.e., we use correction coefficients from the graphic dependence τ_c_^β^/τ_c_^βref^ at β_ref_ = 90° (Figure S18), the best correlation is obtained (Figure 9c).

Thus, we measured 11 distances in the SYN molecule using the calibration method in the isolated spin pair approximation and showed that the relative deviations from the calculated data do not exceed −3.2% (Table S1). The choice of a reference distance when estimating distances turns out to be rather important under conditions of diffusion anisotropy.

#### 2.3.5. Study of Conformational Equilibrium Using NOE

**Figure 10:**
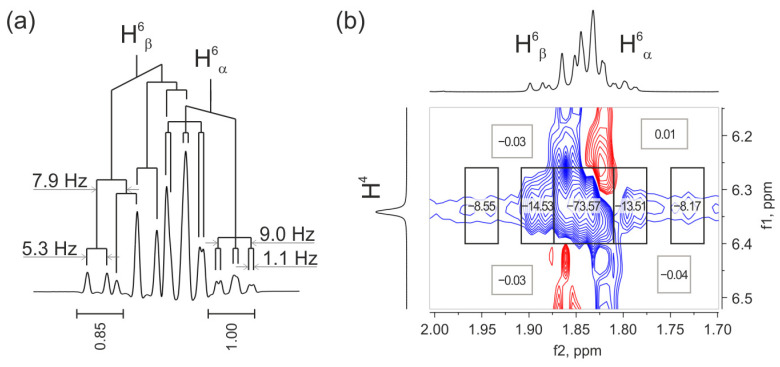
The structure of multiplets for protons H^6^_α_ and H^6^_β_ (**a**) and the fragment of the NOESY spectrum of ANTI containing cross-peaks H^4^/H^6^_α_ and H^4^/H^6^_β_ (**b**). Legend: negative and positive NOE signals are indicated in blue and red, respectively.

The fast conformational exchange in the ANTI molecule should lead to averaging of interproton distances. Their measurement using NOE can serve as independent proof of its existence in solution as a set of conformations [51,52]. A simple comparison of the integrated intensities of some cross-peaks in the NOESY spectrum with the calculated interproton distances for a single conformation reveals inconsistencies that indicate the dynamic nature of the system. However, in this case, the signals of H^6^_α_ и H^6^_β_ protons, the distance between which was used as a reference, largely overlap (Figure 10a).

The difference between the chemical shifts of the proton signals H^6^_α_ and H^6^_β_ is only 0.05 ppm, while the geminal constant is practically comparable to it (−13.6 Hz). In this strongly coupled two-spin AB-type system, the outer components of the AB doublets turn out to be smaller in intensity but equal to each other, and their more intense inner components overlap. The outer components have a different structure of multiplets: for the H^6^_α_ proton, it is a triplet of doublets, and for the H^6^_β_ proton, it is a doublet of doublets, with one of its four components hidden under the intense signal of the inner part of the AB doublet of the H^6^_β_ signal. Despite such overlapping, the outer components were used to determine the NOE ratio of the protons (Figure 10b), although accurate integration, taking into account the contribution of the t_2_ noise band and only three of the four components of the doublet of doublets, was required to accurately determine the experimental cross-peak ratio.

To quantify the ratio of *anti*-conformers, we considered a pair of distances between N^4^H and protons of the C^6^H_2_ group, which turns out to be the most sensitive to changes in the conformation: r(H^4^–H^6^_α_) = 4.25 Å and r(H^4^–H^6^_β_) = 3.62 Å for ANTI1, while r(H^4^–H^6^_α_) = 3.59 Å and r(H^4^–H^6^_α_) = 4.25 Å for ANTI2. We used the formula for the average value of the interproton distance <r> for two-position exchange between states (A) and (B):

(1) $$\langle r\rangle =\sqrt[{6}]{\frac{r_{A}{}^{6}r_{B}{}^{6}}{P_{A}r_{B}{}^{6}+P_{B}r_{A}{}^{6}}}.$$

**Figure 11:**
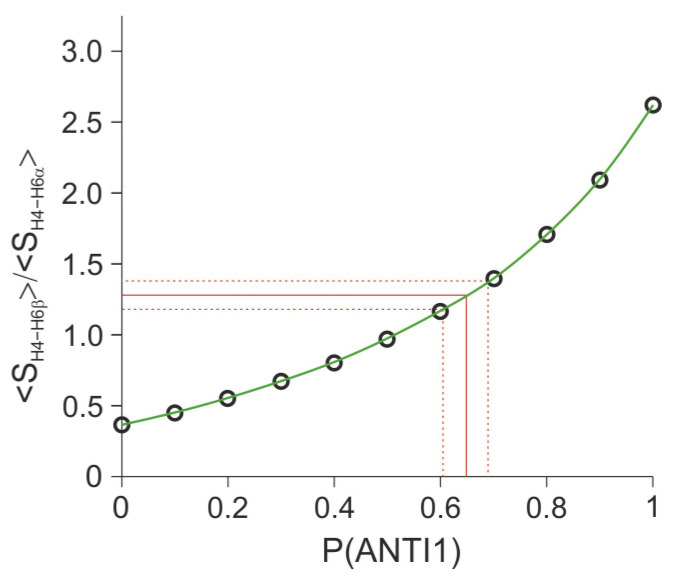
Calculated dependence of the integral intensity ratio of H^4^/H^6^_α_ and H^4^/H^6^_β_ cross-peaks on the ANTI1 conformer population under conditions of fast two-position exchange. Green full line represent the dependence. Red full lines are used to determine the conformer population from the experimental ratio; red dashed lines indicate errors of this value.

Based on the formula above, we plotted the ratio of the average integral intensities of the corresponding cross-peaks <S_H4–H6β_>/<S_H4–H6α_> on the population of the ANTI conformer (Figure 11). The obtained experimental value of this ratio, equal to 1.28, was used to estimate the population ratio of P_A_ (ANTI1) and P_B_ (ANTI2), which is P_A_:P_B_ ≈ 65:35. This result is in good agreement with the data on assessing the conformer ratio using vicinal constants. In this case, the error is about 5% based on the integration error, which, in the vicinity of the measured cross-peaks, did not exceed ±0.2%.

## 3. Materials and Methods

### 3.1. Chemistry

All starting compounds and reagents used are commercially available. The progress of the reactions was monitored by TLC on Silica gel 60 F254 plates (Merck, Darmstadt, Germany) using *n*-hexane/ethyl acetate eluent. Purification and isolation of the products were carried out using an Isolera Four Flash chromatograph on SNAP KP-Sil 100 g cartridges (Biotage, Uppsala, Sweden) with *n*-hexane/ethyl acetate eluent. HPLC analysis was performed on an LC-20 Prominence (Shimadzu, Kyoto, Japan) using a Nucleodur PolarTec column (Macherey-Nagel, Dueren, Germany), length 150 mm, internal diameter 3.0 mm, particle size 3 µm, in acetonitrile–0.1% trifluoroacetic acid (50/50), flow rate 0.4 mL/min, oven temperature 40 °C. Mass spectra were recorded on an LCMS-2020 device (Shimadzu) with a single quadrupole detector under positive mode, electrospray ionization (ESI).

#### 3.1.1. General Procedure for Reduction in Methanol

First, 0.05 g of metallic sodium was added to 50 mL of anhydrous methanol. When the sodium was completely dissolved, 22.8 mmol of methyl or ethyl 5,7-dimethylpyrazolo[1,5-a]pyrimidine-3-carboxylate (**4a** or **4b**) was added at cooling, and then 2.6 g of freshly pounded NaBH_4_ (68.5 mmol, 3 equv.) was added with vigorous stirring. The mixture was stirred for 3 h at room temperature. Methanol was distilled off to dryness, and the residue was diluted with water and extracted with ethyl acetate (3 × 100 mL). The organic layers were dried over Na_2_SO_4_, and the solvent was distilled off under reduced pressure. Yields: 78%—**6a**, 11%—**7a**, and 6%—**5a** for **4a** according to HPLC analysis (Figure S19); 54%—**6b**, 7%—**7b**, and 4%—**5b** for **4b** according to HPLC analysis; low yields are associated with the fact that up to 31% of the reaction products undergo transesterification under the conditions used (Figure S20). The products with different retention factor values can be isolated from the reaction mixture (*R_f_* (**5b**) = 0.64, *R_f_* (**5a**) = 0.53, *R_f_* (**6b**) = 0.30, *R_f_* (**7b**) = *R_f_* (**6a**) = *R_f_* (**6b**) = 0.23; *n*-hexane/ethyl acetate 3:1).

#### 3.1.2. General Procedure for Reduction in Ethanol

First, 0.1 g of metallic sodium was added to 25 mL of anhydrous ethanol. When the sodium was completely dissolved, 8.76 g of ethyl 5,7-dimethylpyrazolo[1,5-a]pyrimidine-3-carboxylate (0.04 mol, **4b**) was added at cooling, and then 4.44 g of freshly pounded NaBH_4_ (0.12 mol, 3 equv.) was added with vigorous stirring. The mixture was stirred for 24 h at 60 °C. The work up was performed similarly to that described above. The mixture of products was separated by flash chromatography. Yields: 71%—**6b**, 10%—**7b**, and 4%—**5b**.

*Ethyl 5,7-dimethylpyrazolo[1,5-a]pyrimidine-3-carboxylate* (**4b**). Colorless solid. ^1^H NMR (400 MHz, DMSO-*d*_6_) δ 8.50 (s, 1H, H^2^), 7.08 (d, *J* = 0.9 Hz, 2H, H^6^), 4.27 (q, *J* = 7.1 Hz, 2H, C^8′^H_2_), 2.69 (d, *J* = 0.9 Hz, 3H, 7-CH_3_), 2.56 (s, 3H, 5-CH_3_), 1.30 (t, *J* = 7.1 Hz, 3H, C^9′^H_3_). ^13^C NMR (101 MHz, DMSO-*d*_6_) δ 162.29 (C^5^), 161.78 (C^8^=O), 147.00 (C^3′^), 146.59 (C^7^), 146.43 (C^2^H), 110.64 (C^6^H), 100.79 (C^3^), 59.33 (C^8′^H_2_), 24.50 (5-CH_3_), 16.53 (7-CH_3_), 14.43 (C^9′^H_3_). MS (ESI) *m/z* (exp.): 220.2 [M + H]^+^; calculated for C_11_H_13_N_3_O_2_ [M + H]^+^: 220.10.

*Ethyl 5,7-dimethyl-4,5-dihydropyrazolo[1,5-a]pyrimidine-3-carboxylate* (**5b**). Colorless viscous liquid. ^1^H NMR (400 MHz, DMSO-*d*_6_) δ 7.47 (s, 1H, H^2^), 6.79 (t, *J* = 2.0 Hz, 1H, NH^4^), 4.91 (dt, *J* = 3.2, 1.5 Hz, 1H, H^6^), 4.29–4.22 (m, 1H, H^5^), 4.16 (q, *J* = 7.1 Hz, 2H, C^8′^H_2_), 2.04 (t, *J* = 1.5 Hz, 3H, 7-CH_3_), 1.24 (t, *J* = 7.1 Hz, 3H, C^9′^H_3_), 1.21 (d, *J* = 6.4 Hz, 3H, 5-CH_3_). ^13^C NMR (101 MHz, DMSO-*d*_6_) δ 162.73 (C^8^=O), 146.76 (C^3′^), 139.26 (C^2^H), 130.84 (C^7^), 106.27 (C^6^H), 92.91 (C^3^), 58.82 (C^8′^H_2_), 46.07 (C^5^H), 24.55 (5-CH_3_), 16.00 (7-CH_3_), 14.52 (C^9′^H_3_). MS (ESI) *m*/*z* (exp.): 222.2 [M + H]^+^; calculated for C_11_H_15_N_3_O_2_ [M + H]^+^: 222.12.

*Ethyl (5R,7S)-, (5S,7R)-dimethyl-4,5,6,7-tetrahydropyrazolo[1,5-a]pyrimidine-3-carboxylate* (**6b**). Colorless viscous liquid. ^1^H NMR (400 MHz, DMSO-*d*_6_) δ 7.45 (s, 1H, H^2^), 6.11 (d, *J* = 2.2 Hz, 1H, NH^4^), 4.16 (dq, *J* = −10.8, 7.1 Hz, 1H, C^8′^HH), 4.13 (dq, *J* = −10.8, 7.1 Hz, 1H, C^8′^HH), 4.10 (dqd, *J* = 11.1, 6.4, 4.2 Hz, 1H, H^7^), 3.49 (dqd, *J* = 11.1, 6.4, 2.9 Hz, 1H, H^5^), 2.16 (dddd, *J* = −13.4, 4.2, 2.9, 2.2 Hz, 1H, H^6^_β(eq)_), 1.44 (dt, *J* = −13.4, 11.1, Hz, 1H, H^6^_α(ax)_), 1.42 (d*, J* = 6.4 Hz, 3H, 7-CH_3_), 1.23 (d*, J* = 6.4 Hz, 3H, 5-CH_3_), 1.22 (t, *J* = 7.1 Hz, 3H, C^9′^H_3_). ^13^C NMR (101 MHz, DMSO) δ 163.06 (C^8^=O), 148.18 (C^3′^), 138.20 (C^2^H), 92.96 (C^3^), 58.58 (C^8′^H_2_), 50.51 (C^7^H), 45.02 (C^5^H), 38.64 (C^6^H_2_), 21.01 (5-CH_3_), 19.55 (7-CH_3_), 14.55 (C^9′^H_3_). MS (ESI) *m*/*z* (exp.): 224.2 [M + H]^+^; calculated for C_11_H_17_N_3_O_2_ [M + H]^+^: 224.14.

*Ethyl (5R,7R)-, (5S,7S)-dimethyl-4,5,6,7-tetrahydropyrazolo[1,5-a]pyrimidine-3-carboxylate* (**7b**). Colorless viscous liquid. ^1^H NMR (400 MHz, DMSO-*d*_6_) δ 7.45 (s, 1H, H^2^), 6.33 (dd, *J* = 1.6, 1.1 Hz, 1H, NH^4^), 4.10 (qdd, *J* = 6.4, 5.3, 4.8, Hz, 1H, H^7^), 4.14 (q, *J* = 7.1 Hz, 2H, C^8′^H_2_), 3.59 (dqdd, *J* = 7.9, 6.4, 4.2, 1.6 Hz, 1H, H^5^), 1.86 (ddd, *J* = −13.6, 7.9, 5.3 Hz, 1H, H^6^_β_), 1.81 (dddd, *J* = −13.6, 4.8, 4.2, 1.1 Hz, 1H, H^6^_α_), 1.37 (d*, J* = 6.4 Hz, 3H, 7-CH_3_), 1.23 (t*, J* = 7.1 Hz, 3H, C^9′^H_3_), 1.21 (d, *J* = 6.4 Hz, 3H, 5-CH_3_). ^13^C NMR (101 MHz, DMSO) δ 162.99 (C^8^=O), 147.36 (C^3′^), 138.48 (C^2^H), 92.51 (C^3^), 58.49 (C^8′^H_2_), 48.39 (C^7^H), 41.52 (C^5^H), 35.03 (C^6^H_2_), 21.14 (5-CH_3_), 21.08 (7-CH_3_), 14.55 (C^9′^H_3_). MS (ESI) *m*/*z* (exp.): 224.2 [M + H]^+^; calculated for C_11_H_17_N_3_O_2_ [M + H]^+^: 224.14.

#### 3.1.3. General Procedure for Reduction with Tetrabutylammonium Borohydride

According to [53], a solution of tetrabutylammonium borohydride (4 equiv.) and ethyl 5,7-dimethylpyrazolo[1,5-a]pyrimidine-3-carboxylate (1 equiv.) in chloroform was heated at reflux for 24 h. Then, the reaction mixture was quenched by the addition of 3% aqueous hydrogen peroxide and 10% aqueous sodium hydroxide. The mixture was stirred for 2 h, the layers were separated, and the organic layer was analyzed by HPLC (Figure S21).

### 3.2. Calculations

All calculations were performed on an HP Z8G4 workstation (nVidia Quadro RTX 5000) using Schrodinger Suite 2020-4. The initial geometry of stereoisomers was obtained using MacroModel 10.8. The conformational search was carried out by the method of Torsional sampling molecular mechanics (MCMM) in the OPLS force field. The resulting conformers were optimized using Jaguar 9.5. Optimization was carried out according to the density functional theory using the Becke–Lee–Yang–Parr hybrid exchange–correlation functional and the 6-31G** basis set (DFT B3LYP/6-31G**), the PBF solvation model, and the solvent DMSO. The optimized structures were ranked according to the potential energy value, and the obtained absolute energy values were converted into relative energy values (QM Relative Energy).

### 3.3. NMR Studies

All 1D and 2D NMR spectra were recorded in DMSO-*d*_6_ on an Avance III HD 400 MHz spectrometer (Bruker) with inverse probe equipped with Z-gradient. All measurements were carried out without sample spinning at 295 K. Initial time-domain signals (FID) were processed in the MestReNova 12.0.0 program. Routine 1D data were apodized with 0.5 Hz exponential functions, while 2D data were treated with 90 degree sine squared (t_1_) and 90 degree sine bell (t_2_) functions prior to Fourier transform.

Experimental vicinal constants were determined directly from ^1^H spectrum (SW = 4 kHz), which was obtained as a result of the following additional processing of the free induction decay signal (32,768 data points, digital resolution 0.122 Hz/pt): Lorentz–Gauss transformation (LB = −4 Hz, GB = 2.0 Hz), zero filling up to 256 K, and direct linear prediction (LP) using 24 coefficients. This procedure provided a digital resolution of 0.015 Hz/pt and an accuracy of the vicinal constants determining no worse than ±0.02 Hz. The values of the calculated scalar constants were determined using the MestRe-J 1.1 program. In all cases, the HLA modification of the Karplus equation for the ethane fragment, which considers the electronegativity of atoms in α- and β-positions (HLA with β-effect), was used [54].

Common acquisition parameters for phase-sensitive 2D-NOESY spectra of the studied compounds were the following: 1024 t_2_ points, 200–256 t_1_ points, 3.6 kHz sweep width, and 0.3 s t_2_ acquisition time (pulse sequence NOESYgpph). To find the maximal signal-to-noise ratio for most cross-peaks and to check the implementation of the isolated spin pair approximation (ISPA), 2D-NOESY spectra were recorded at different relaxation delays of 1–5 s and varied mixing times of 0.3–1.2 s. The best result was registered at D_1_ = 5.0 s and τ_m_ = 0.5 s. All 2D-NOESY spectra were obtained without zero-quantum coherence elimination and processing procedures, such as base plane correction and decreasing t_1_ noise. Nonoverlapping cross-peaks, including low-intensity ones, were integrated by the rectangle boxing method, which gives more reliable results, especially in the case of a strong t_2_ noise band. A PANIC (peak amplitude normalization for improved cross-relaxation) approach was used at the quantitative level of estimating interproton distances.

## 4. Conclusions

As a result of this study, it was first demonstrated that both *syn*- and *anti*-isomers are formed upon reduction of 5,7-substituted pyrazolo[1,5-a]pyrimidines with borohydrides, while their ratio depends on the reaction conditions and the reducing agent used. Detailed NMR studies not only confirmed the structure of the target products of this reaction, but also showed significant differences between isomers in terms of conformational behavior. While the *syn*-configured product has a rigid scaffold, it was used to demonstrate the possibility of using long-range NOEs to quantify interproton distances. Using the SYN molecule as an example, a significant effect of t_1_ and t_2_ noise in the NOESY spectrum on the accuracy of estimating long-range interproton distances (>4.0 Å) was demonstrated. A procedure for integrating cross-peaks and their nearest neighborhoods, which allows one to significantly reduce the contribution of artifacts, is proposed. The developed procedure allowed us to estimate long-range interproton distances (from 4 to 6.5 Å) with an accuracy of about 5%, which was not previously performed for small molecules.

In turn, the *anti*-configured product is the dynamic system capable of reorienting its substituents at positions five and seven, thereby fine tuning to the active site of the target. Based on the analysis of calculated and experimental data, it was established that in solution, the ANTI molecule is subjected to fast transitions between two conformations due to the inversion of the pyrimidine ring of the scaffold. The equilibrium ratio of ANTI conformers was estimated based on the analysis of vicinal constants and NOESY data, showing very close values.

The results of this study indicate the high potential of *anti*-configured 5,7-substituted tetrahydropyrazolo[1,5-a]pyrimidines for the development of selective drug candidates. However, both studied 5,7-substituted tetrahydropyrazolo[1,5-a]pyrimidines are interesting objects in terms of NMR studies. The set of properties—rigidity and mobility—is unique when considering structures of the same chemotype.

## Figures and Tables

**Table 1:** Calculated values of dihedral angles, vicinal constants, and their experimental values for SYN.

N	H_i_–H_j_	Θ_ij_^calc^, Grad	^3^*J*_ij_^calc^, Hz	^3^*J*_ij_^exp^, Hz
1	H^6^_α(ax)_–H^5^	−177.7	11.85	11.1
2	H^6^_α(ax)_–H^7^	168.8	11.32	11.1
3	H^6^_β(eq)_–H^5^	−60.2	2.64	2.9
4	H^6^_β(eq)_–H^7^	51.7	4.13	4.2

**Table 2:** Calculated values of dihedral angles, vicinal constants, and their experimental values for ANTI1 and ANTI2.

N	H_i_–H_j_	Θ_ij_^calc^, Grad		^3^*J*_ij_^calc^, Hz		<^3^*J*_ij_>^exp^, Hz
		ANTI1	ANTI2	ANTI1	ANTI2	
1	H^6^_α_–H^5^	59.6	−52.3	2.86	4.41	4.2
2	H^6^_α_–H^7^	76.1	166.5	0.87	11.88	4.8
3	H^6^_β_–H^5^	176.7	65.0	11.85	1.95	7.9
4	H^6^_β_–H^7^	−41.2	49.7	6.32	4.46	5.3

## Data Availability

Not applicable.

## Supplementary Materials

The following supporting information can be downloaded at: https://www.mdpi.com/article/10.3390/molecules28186584/s1, Table S1: Experimental (NOE) and calculated distance values (Å) for **6b** (SYN). Figure S1: The structure of the 5,7-dimethyl-4,5,6,7-tetrahydropyrazolo[1,5-a]pyrimidine-3-carboxylate molecule with atom numbering. Figure S2: ^1^H NMR spectrum of **4b**. Figure S3: ^13^C NMR spectrum of **4b**. Figure S4: ^1^H NMR spectrum of **5b**. Figure S5: ^13^C NMR spectrum of **5b**. Figure S6: ^1^H NMR spectrum of **6b** (SYN). Figure S7: Magnified non-singlet signals in ^1^H NMR spectrum of **6b** (SYN). Figure S8: ^13^C NMR spectrum of **6b** (SYN). Figure S9: COSY spectrum of **6b** (SYN). Figure S10: HSQC spectrum of **6b** (SYN). Figure S11: NOESY spectrum of **6b** (SYN). Figure S12: ^1^H NMR spectrum of **7b** (ANTI). Figure S13: Magnified non-singlet signals in ^1^H NMR spectrum of **7b** (ANTI). Figure S14: ^13^C NMR spectrum of **7b** (ANTI). Figure S15: COSY spectrum of **7b** (ANTI).Figure S16: HSQC spectrum of **7b** (ANTI). Figure S17: NOESY spectrum of **7b** (ANTI). Figure S18: Illustration of estimating the contribution of diffusion anisotropy (in %) to measured distances at the diffusion anisotropy parameter A = 4.4 for **6b** (SYN). Figure S19: HPLC analysis of product mixture obtained from reduction of **4a** in methanol. Figure S20: HPLC analysis of product mixture obtained from reduction of **4b** in methanol. Figure S21: HPLC analysis of reaction mixture from reduction of **4b** in chloroform. Figure S22: LCMS data of **4b**. Figure S23: LCMS data of **5b**. Figure S24: LCMS data of **6b** (SYN). Figure S25: LCMS data of **7b** (ANTI).

## Sample Availability

Samples of the compounds **4b**, **5b**, **6b**, and **7b** are available from the authors upon request.
