# Supplementary material for: NMR-Verified Dearomatization of 5,7-Substituted Pyrazolo[1,5-a]pyrimidines
Source: Molecules. 2023 Sep 12;28(18):6584. doi: 10.3390/molecules28186584 (PMC10535613; doi:10.3390/molecules28186584)
Supplement: Supplementary file 1 [file molecules-28-06584-s001.zip › molecules-2576597-supplementary.pdf]

Supplementary Materials

NMR-Verified Dearomatization of 5,7-Substituted Pyrazolo[1,5-a]pyrimidines

Daria Novikova [1,*], Ammar Al Mustafa [1], Tatyana Grigoreva [1], Svetlana Vorona [1], Stanislav Selivanov [2,3] and Vyacheslav Tribulovich [1,*]

[1] Laboratory of Molecular Pharmacology, St. Petersburg State Institute of Technology, St. Petersburg 190013, Russia; ammar.almus@hotmail.com (A.A.M.); rozentatiana@gmail.com (T.G.); s.vorona@bk.ru (S.V.)
[2] Laboratory of Biomolecular NMR, St. Petersburg State University, St. Petersburg 199034, Russia; nmr.group.spbu@gmail.com
[3] Department of Organic Chemistry, St. Petersburg State Institute of Technology, St. Petersburg 190013, Russia
* Correspondence: dc.novikova@gmail.com (D.N.); tribulovich@gmail.com (V.T.)

Table S1. Experimental (NOE) and calculated distance values (Å) for **6b** (SYN).

N	H_i–H_j	S_{ij}/S_{ii}	r_{exp}^{a}, Å	r_{exp}^{b}, Å	r_{exp}^{c}, Å	r_{calc}, Å	$\Delta r/r^{a}$, %	$\Delta r/r^{b}$, %	$\Delta r/r^{c}$, %
1	$H^6_{\alpha(ax)}$–$H^6_{\beta(eq)}$	15.5	1.77	1.75	1.75	1.77	0	-1.13	-1.13
2	$H^6_{\alpha(ax)}$–H^5	0.58	3.06	3.02	3.04	3.06	0	-1.31	-0.65
3	$H^6_{\alpha(ax)}$–H^7	0.54	3.10	3.06	3.08	3.06	1.31	0	0.65
4	$H^6_{\beta(eq)}$–H^5	2.32	2.39	2.40	2.40	2.48	-3.63	-3.23	-3.23
5	$H^6_{\beta(eq)}$–H^7	2.40	2.37	2.39	2.39	2.43	-2.47	-1.65	-1.65
6	H^2–H^4	0.04	4.78	4.72	4.91	4.95	-3.43	-4.65	-0.81
7	H^2–H^5	0.014	5.69	5.62	5.90	5.85	-2.73	-3.93	0.85
8	H^2–$H^6_{\beta(eq)}$	0.01	6.02	5.95	6.54	6.54	-7.95	-9.02	0
9	H^4–H^5	1.87	2.52	2.49	2.49	2.56	-1.56	-2.73	-2.73
10	H^4–$H^6_{\beta(eq)}$	0.104	4.07	4.03	4.23	4.25	-4.24	-5.18	-0.47
11	H^5–H^7	1.17	2.72	2.69	2.69	2.69	1.12	0	0

^{a}—the reference distance r(H^6_{α}–H^6_{β}); ^{b}—the reference distance r(H^5–H^7); ^{c}—the reference distance r(H^5–H^7) with a correction for diffusion anisotropy.

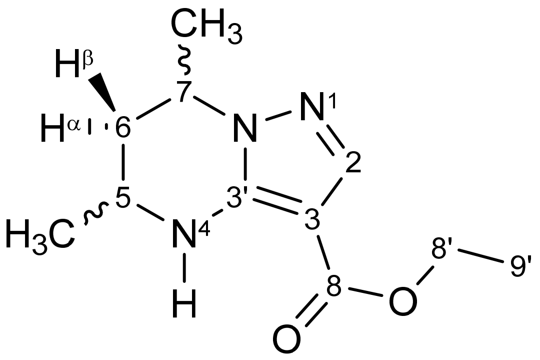

Figure S1. The structure of the 5,7-dimethyl-4,5,6,7-tetrahydropyrazolo[1,5-a]pyrimidine-3-carboxylate molecule with atom numbering.

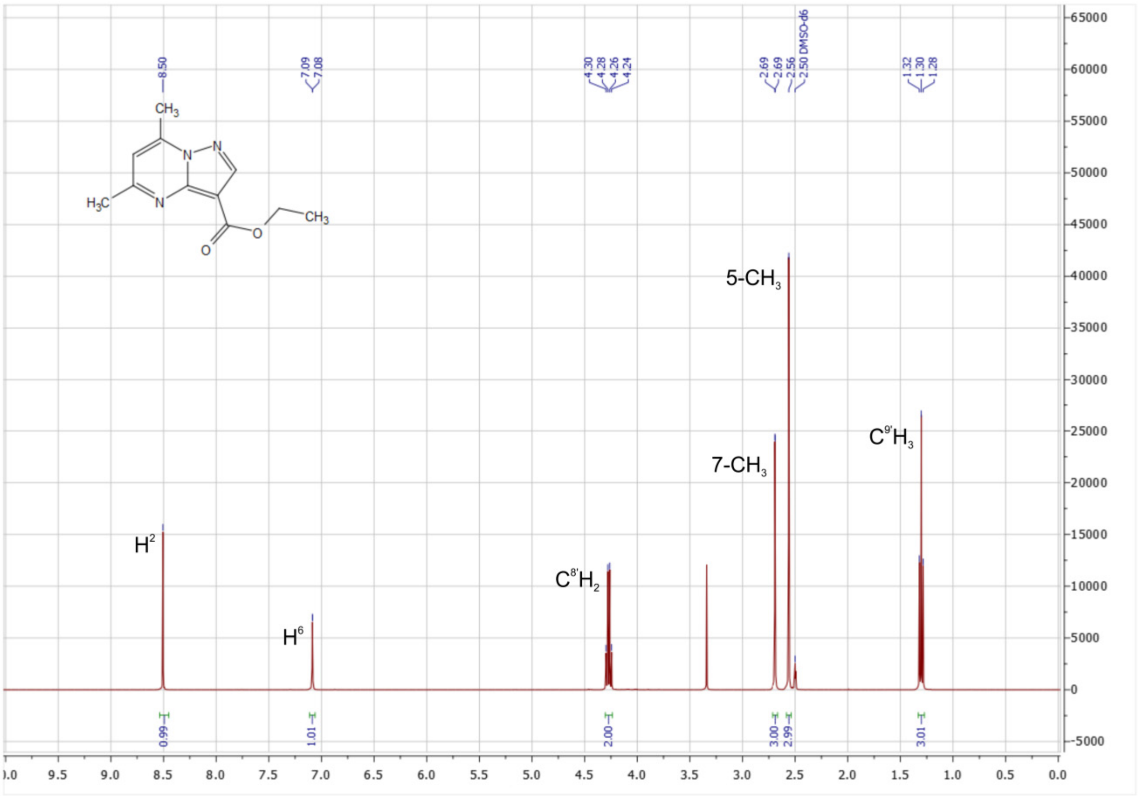

Figure S2. ^{1}H NMR spectrum of **4b**.

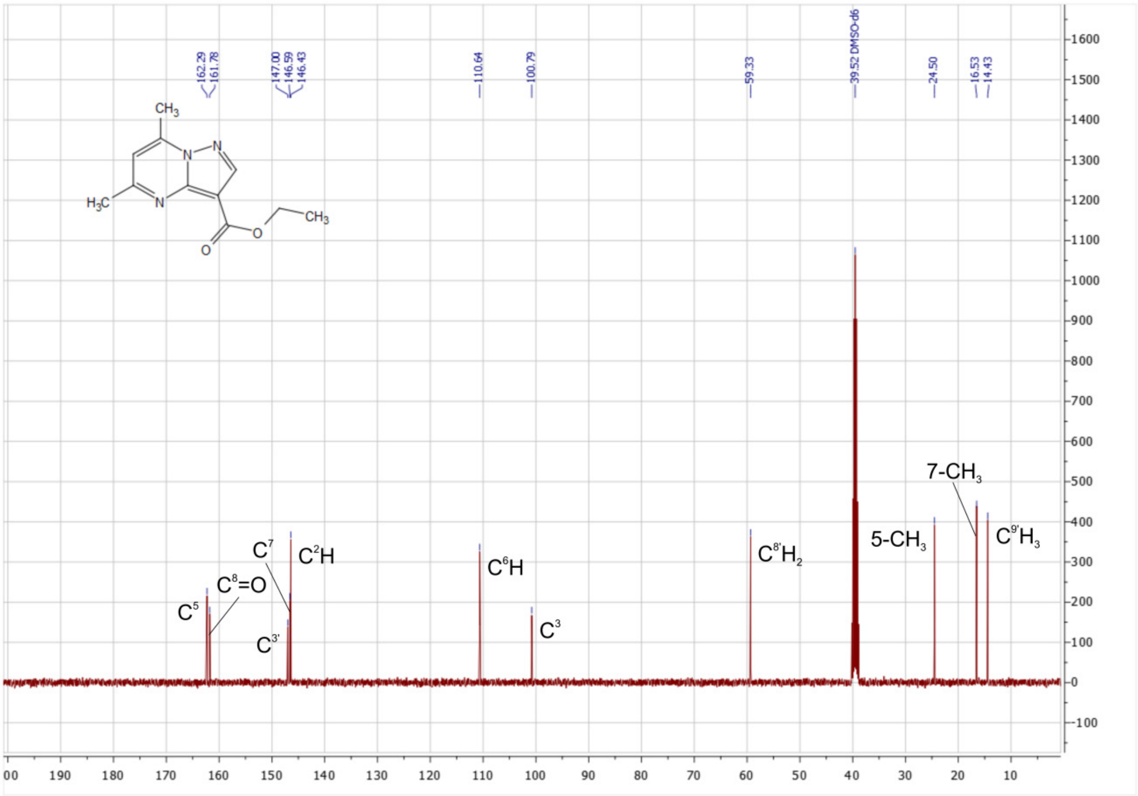

Figure S3. ^{13}C NMR spectrum of **4b**.

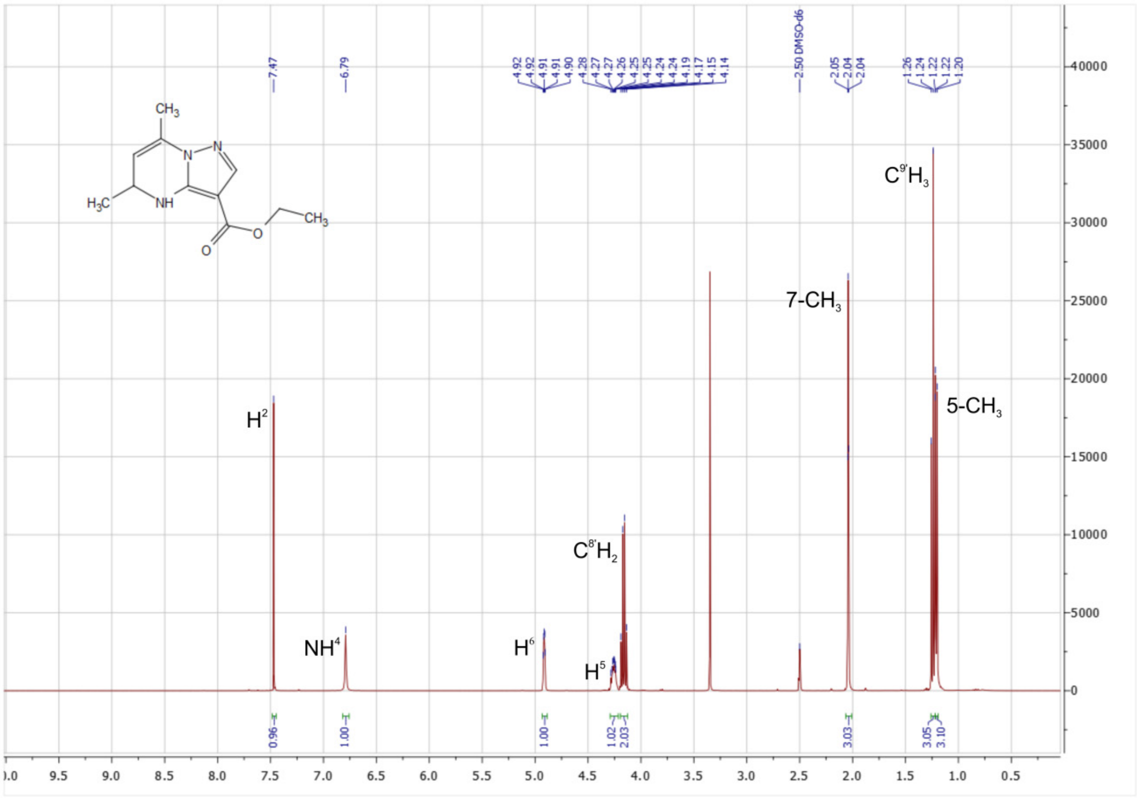

Figure S4. ^{1}H NMR spectrum of **5b**.

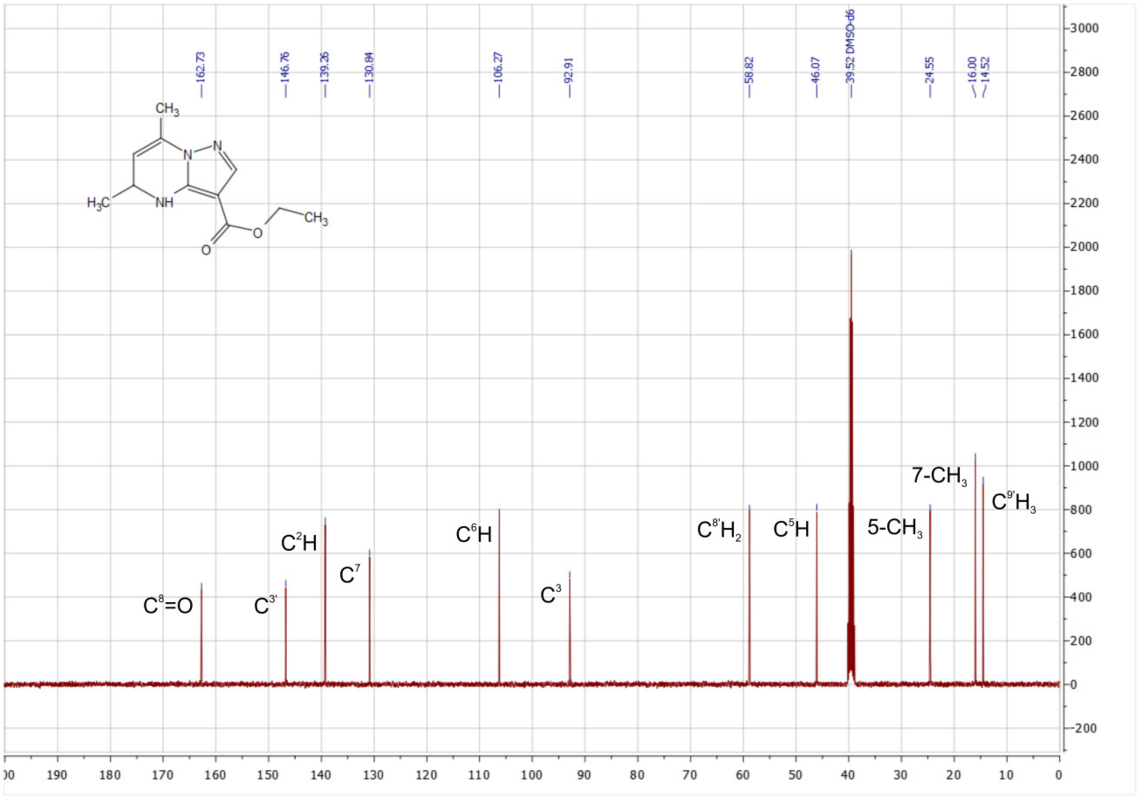

Figure S5. ^{13}C NMR spectrum of **5b**.

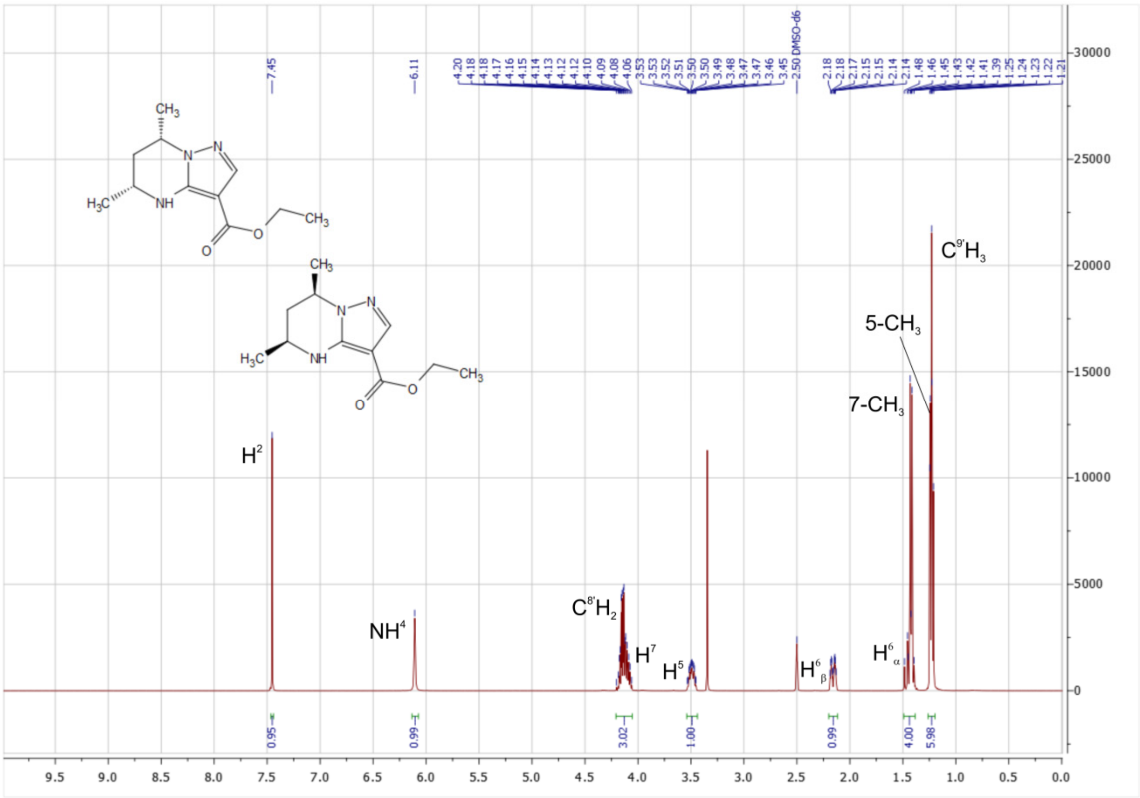

Figure S6. ^{1}H NMR spectrum of **6b** (SYN).

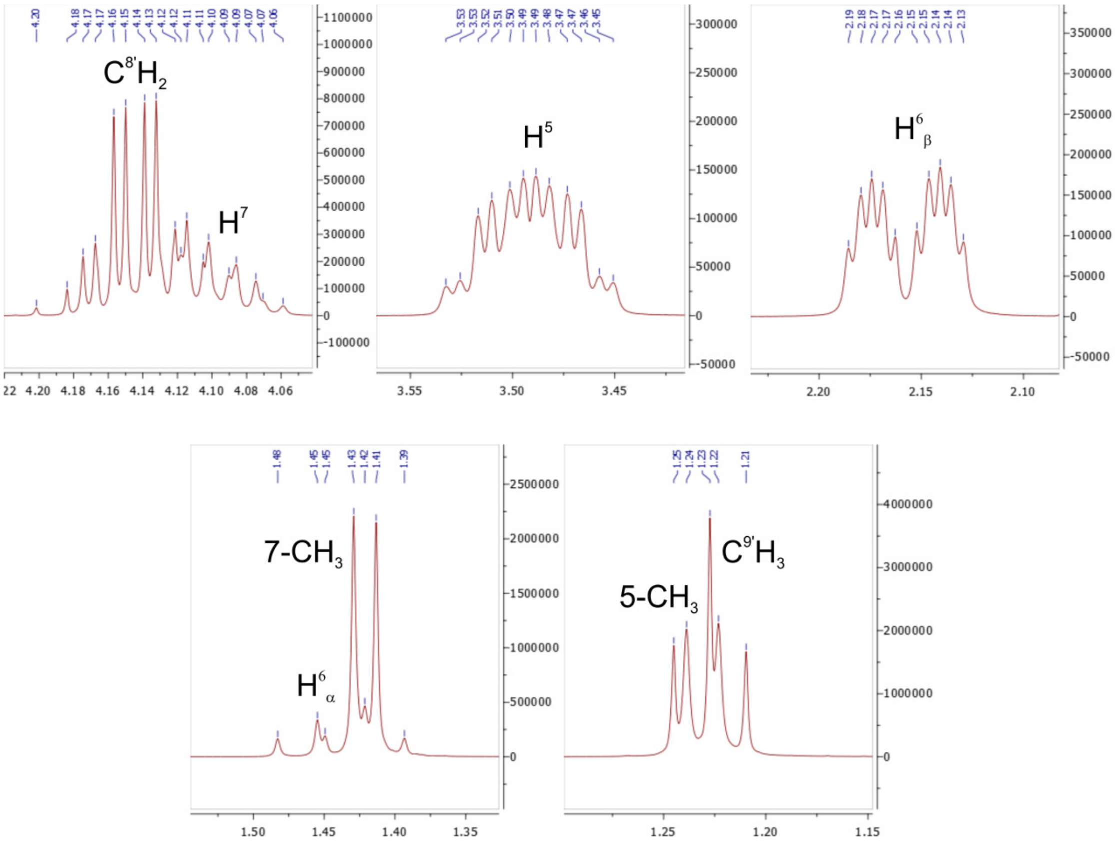

Figure S7. Magnified non-singlet signals in ^{1}H NMR spectrum of **6b** (SYN).

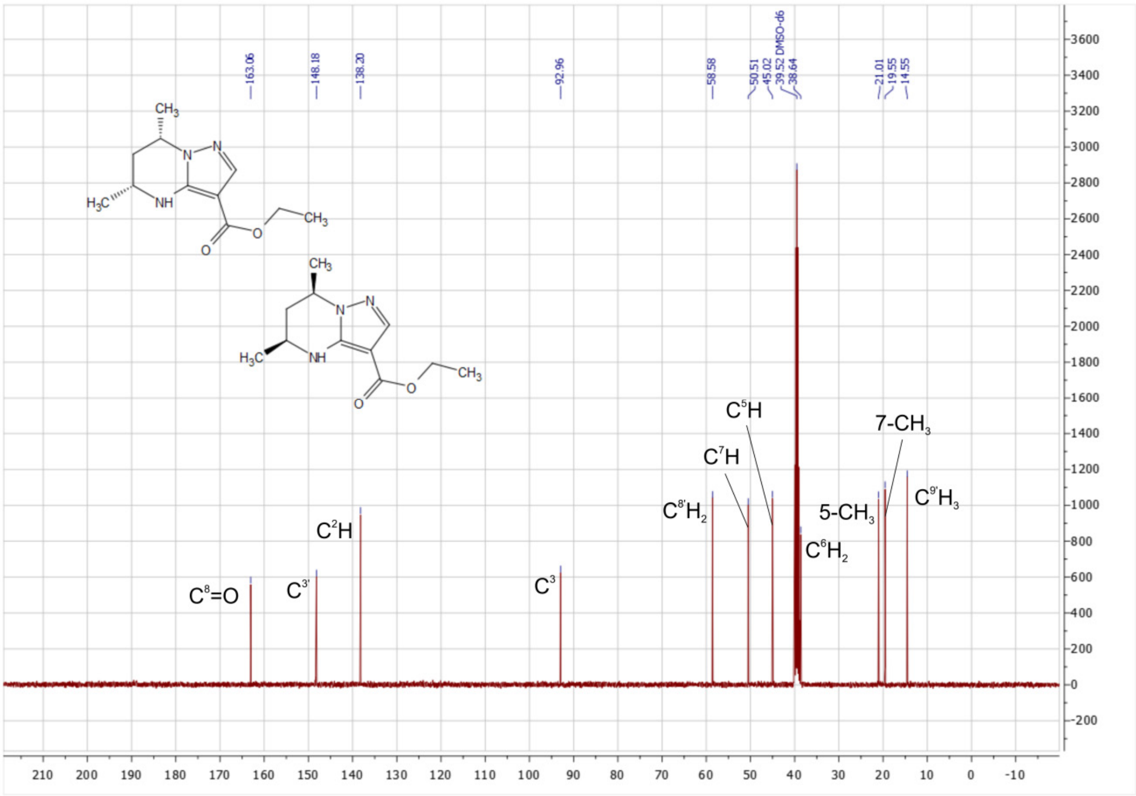

Figure S8. ^{13}C NMR spectrum of **6b** (SYN).

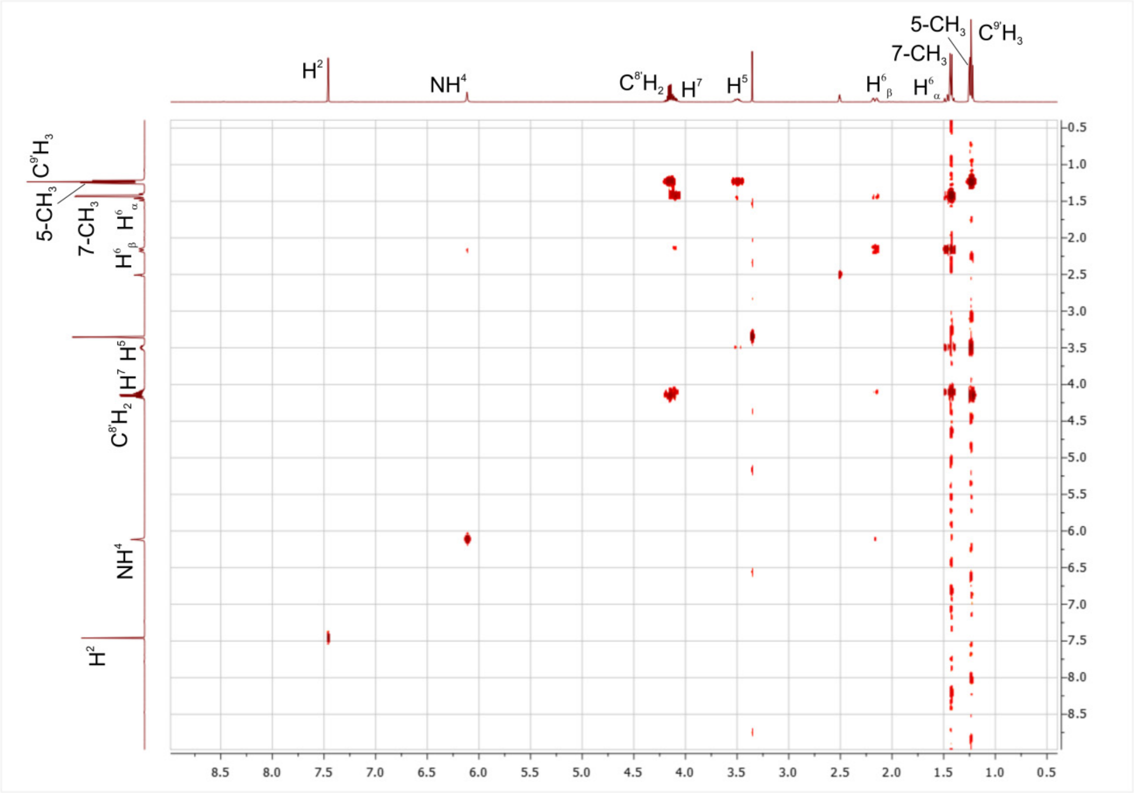

Figure S9. COSY spectrum of **6b** (SYN).

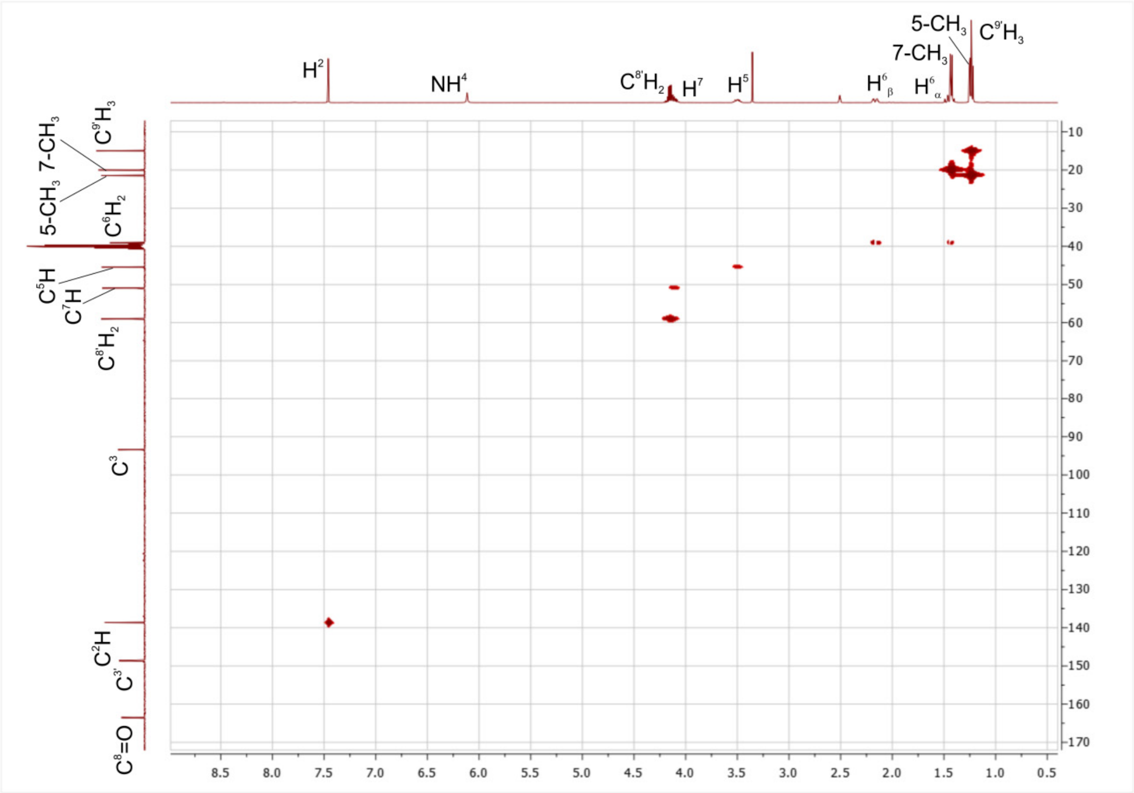

Figure S10. HSQC spectrum of **6b** (SYN).

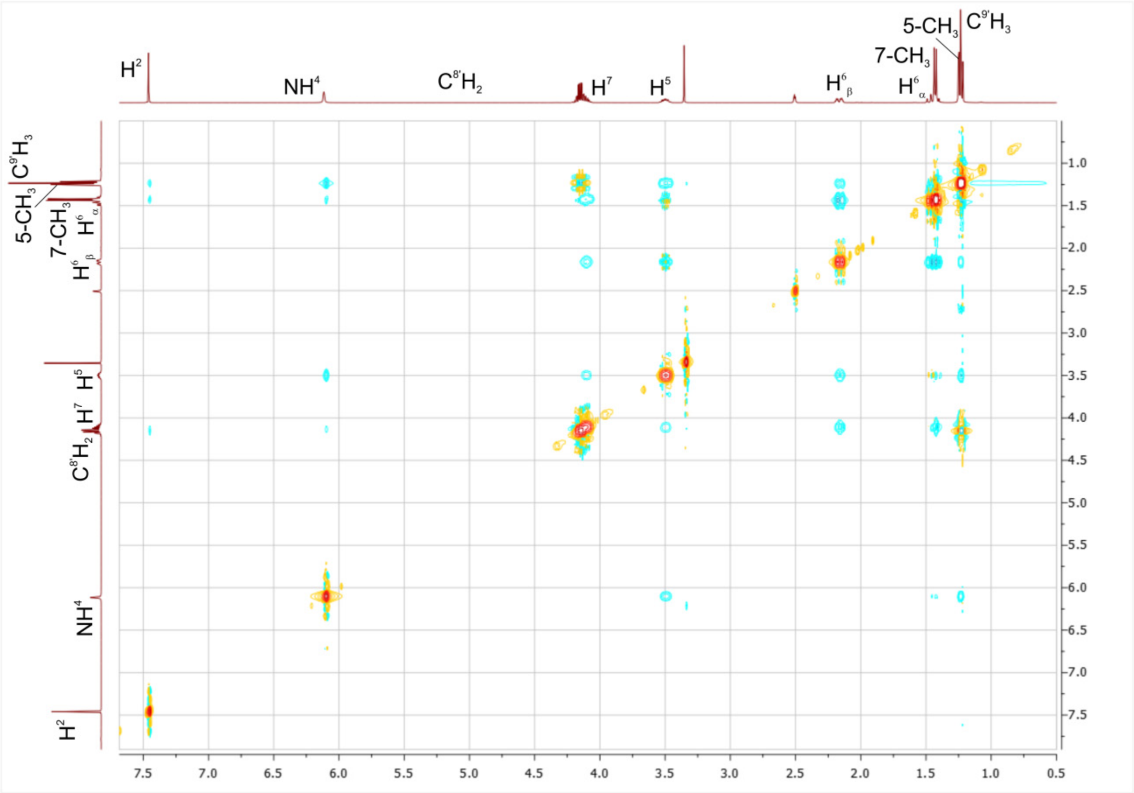

Figure S11. NOESY spectrum of **6b** (SYN).

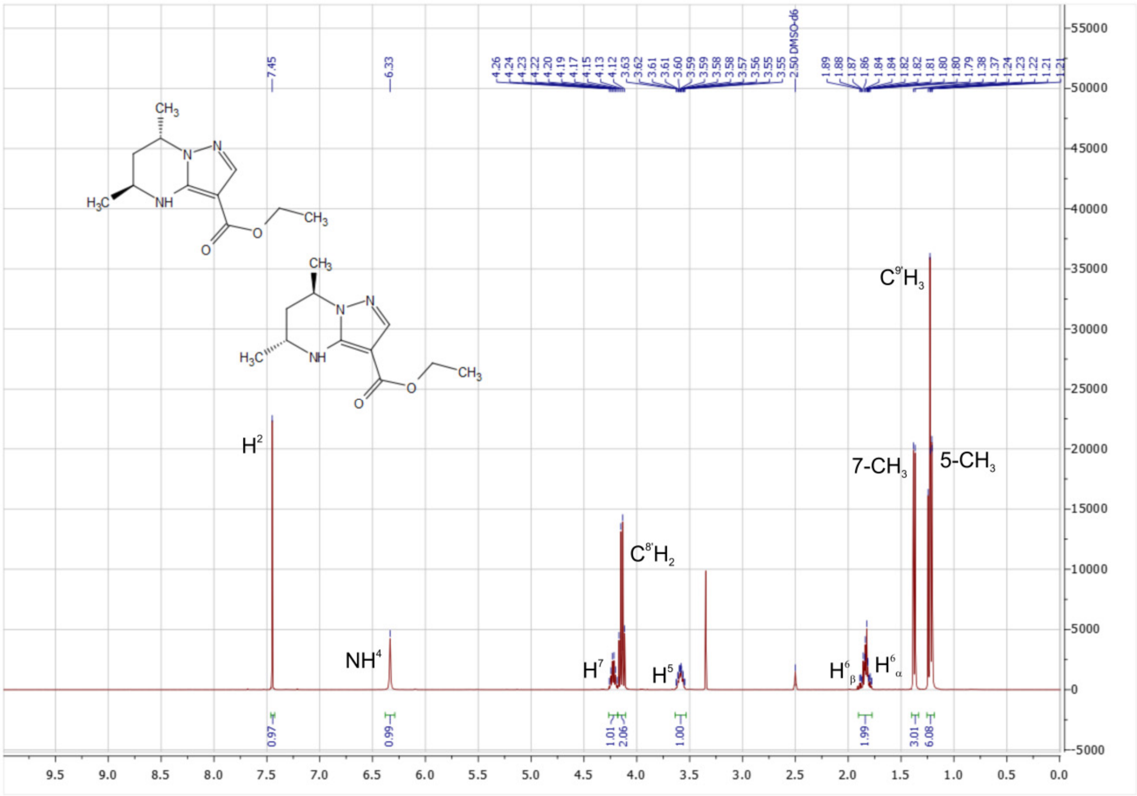

Figure S12. ^{1}H NMR spectrum of **7b** (ANTI).

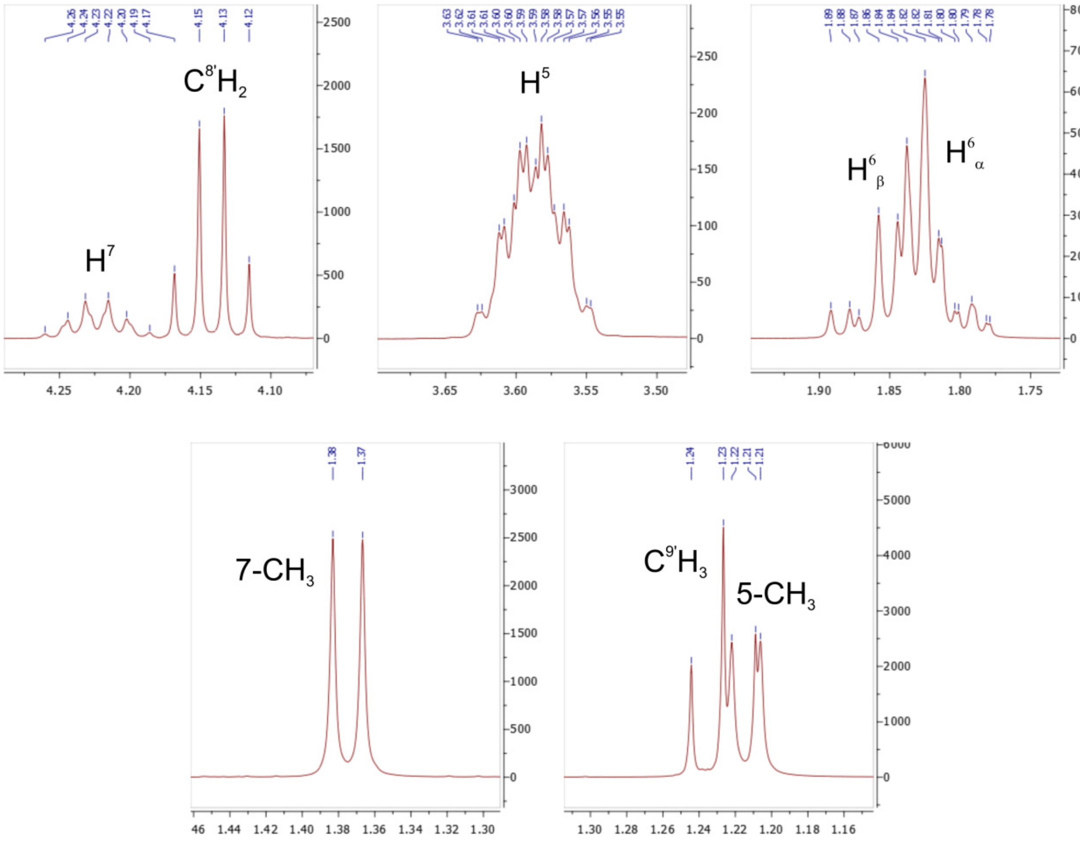

Figure S13. Magnified non-singlet signals in ^{1}H NMR spectrum of **7b** (ANTI).

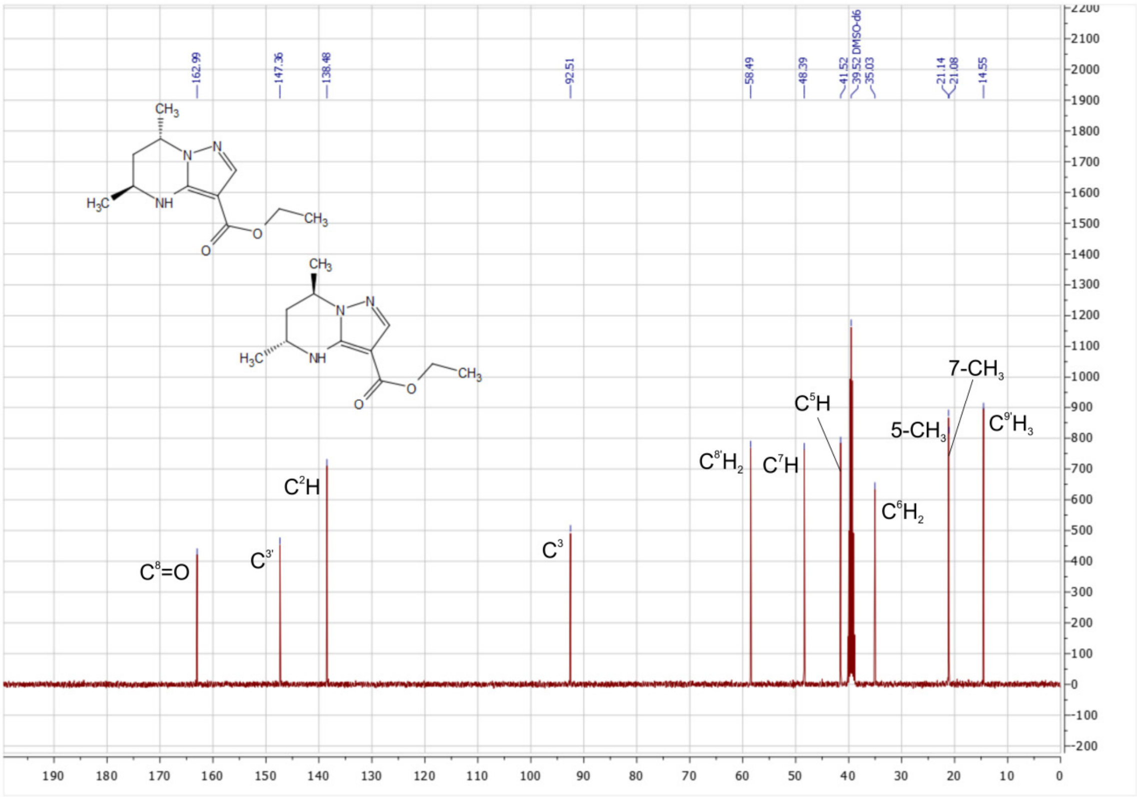

Figure S14. ^{13}C NMR spectrum of **7b** (ANTI).

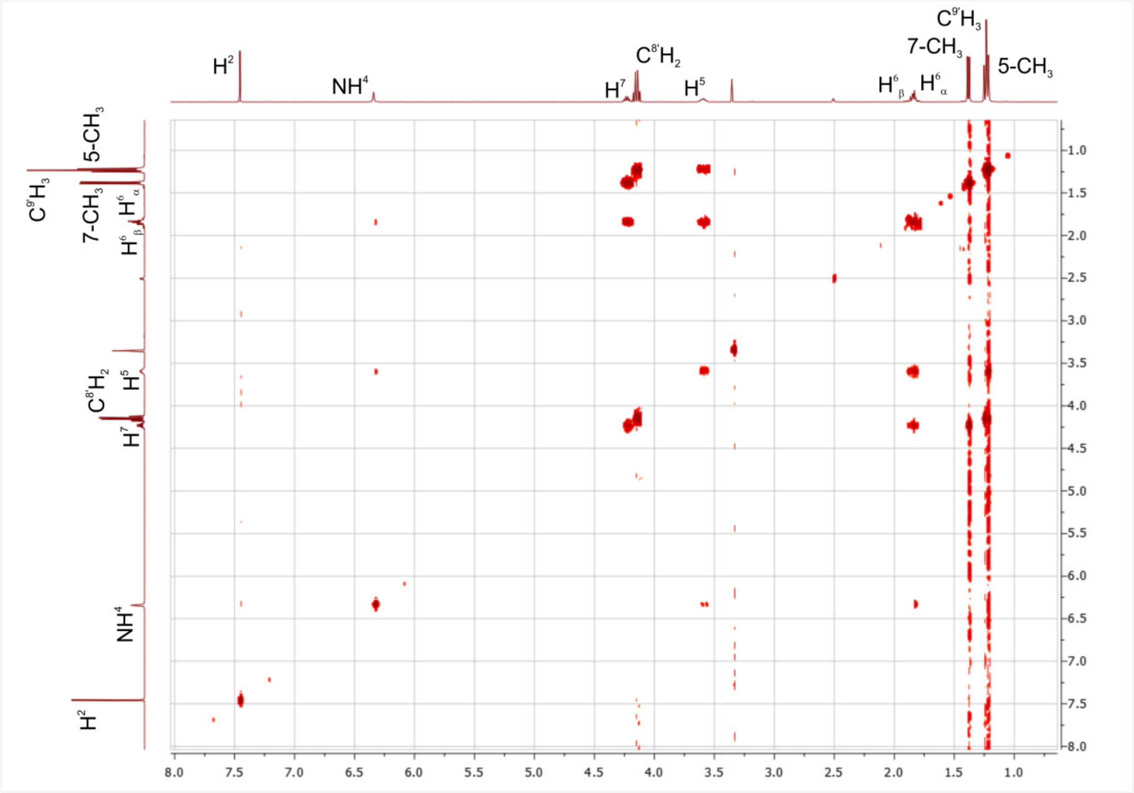

Figure S15. COSY spectrum of **7b** (ANTI).

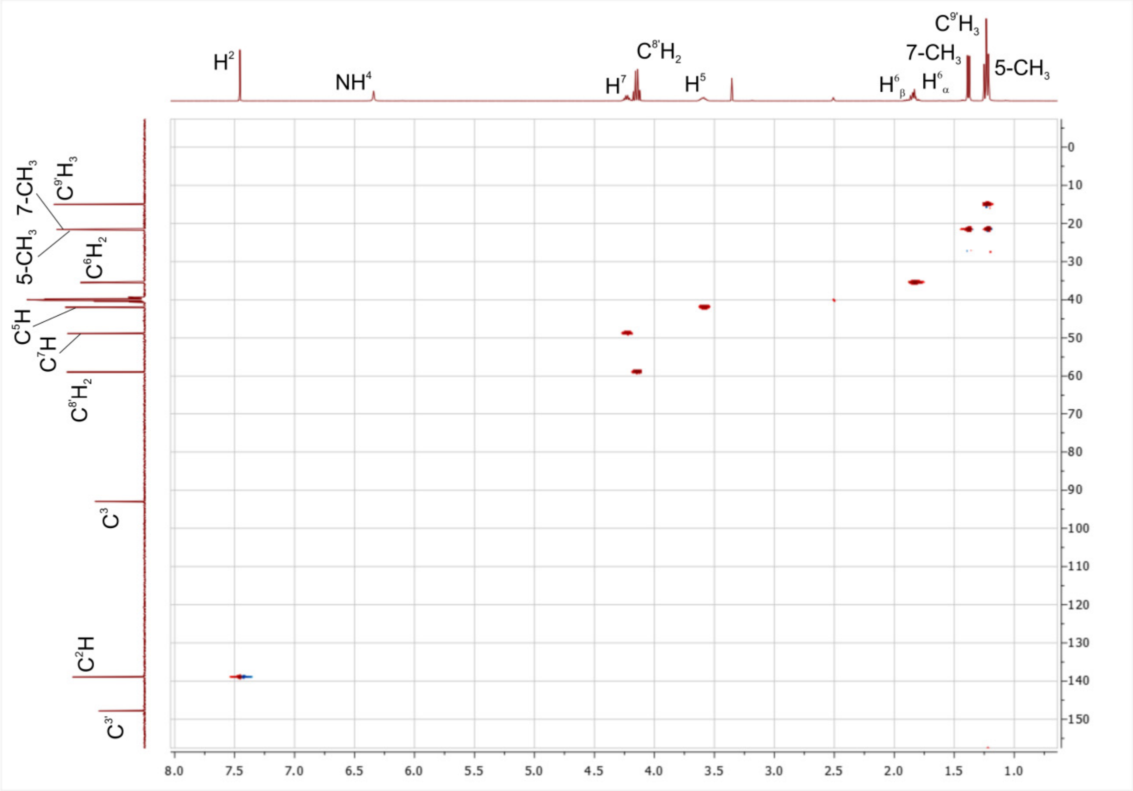

Figure S16. HSQC spectrum of **7b** (ANTI).

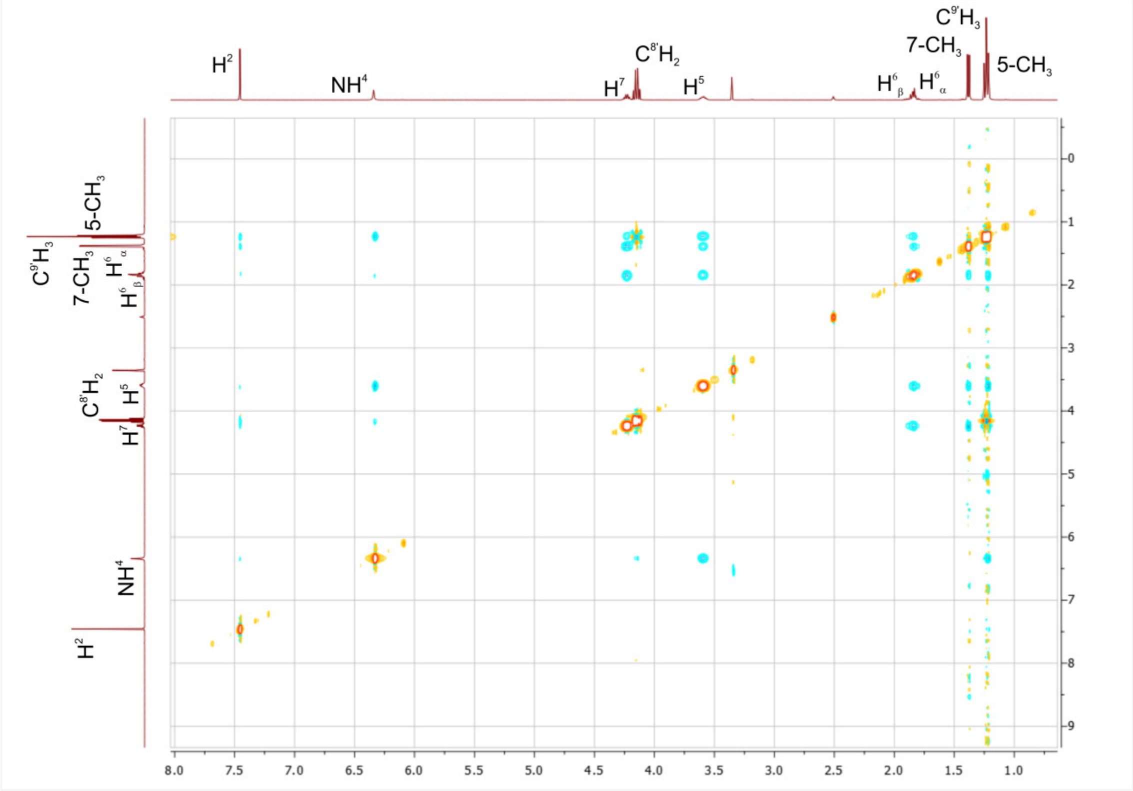

Figure S17. NOESY spectrum of **7b** (ANTI).

$$\frac{\tau_c(\beta)}{\tau_c^{ref}(90)} = \frac{9(A-1)^2}{(A+5)^2}\,Cos^4\beta - \frac{6(A-1)(A-4)}{(A+5)^2}\,Cos^2\beta + 1$$

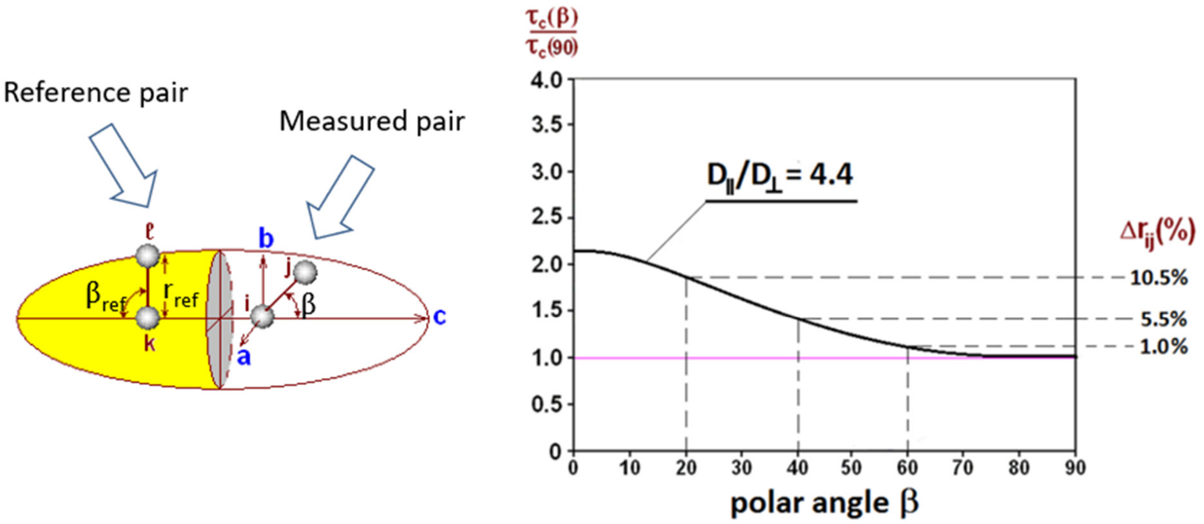

Figure S18. Illustration of estimating the contribution of diffusion anisotropy (in %) to measured distances at the diffusion anisotropy parameter A = 4.4 for **6b** (SYN).

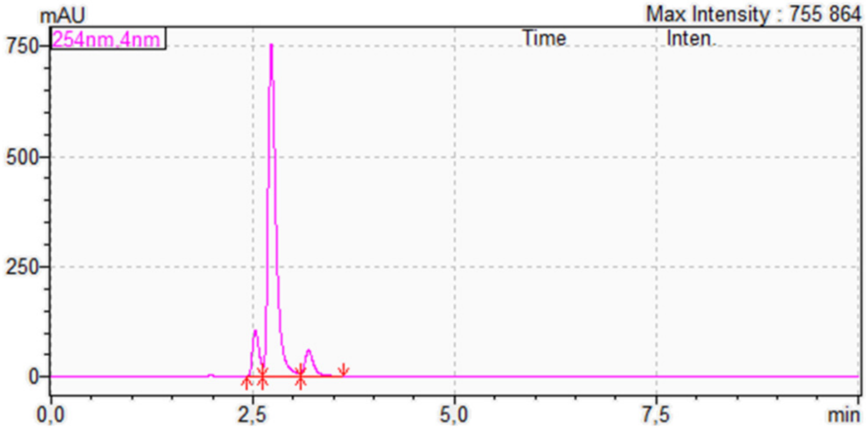

Peak	Compound	Ret. Time	Area	Area %	Height	Height %
1	**7a**	2.530	604083	9.737	104058	11.350
2	**6a**	2.728	5149722	83.005	752580	82.086
3	**5a**	3.189	450284	7.258	60187	6.565
Total			6204089	100.000	916825	100.000

Figure S19. HPLC analysis of product mixture obtained from reduction of **4a** in methanol.

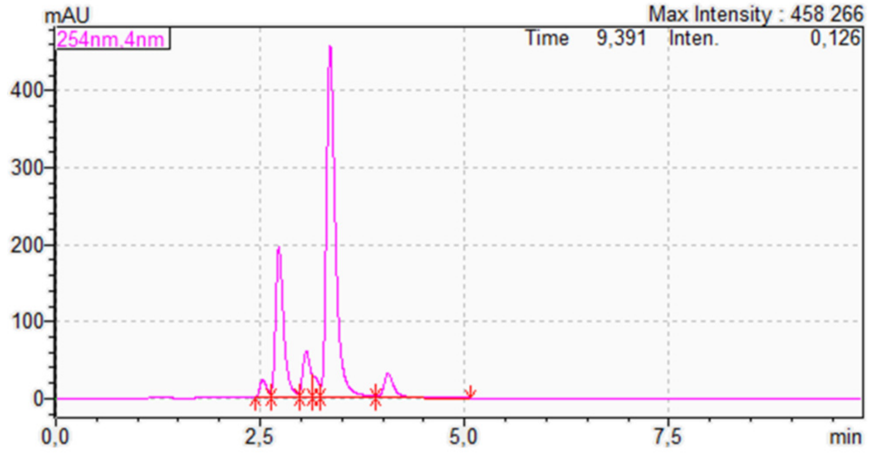

Peak	Compound	Ret. Time	Area	Area %	Height	Height %
1	**7a**	2.531	143894	2.473	23273	2.927
2	**6a**	2.728	1351788	23.233	195161	24.547
3	**7b**	3.063	372462	6.401	60191	7.571
4	**5a**	3.145	143934	2.474	28461	3.580
5	**6b**	3.356	3519322	60.485	456376	57.403
6	**5b**	4.064	287102	4.934	31575	3.972
Total			5818504	100.000	795038	100.000

Figure S20. HPLC analysis of product mixture obtained from reduction of **4b** in methanol.

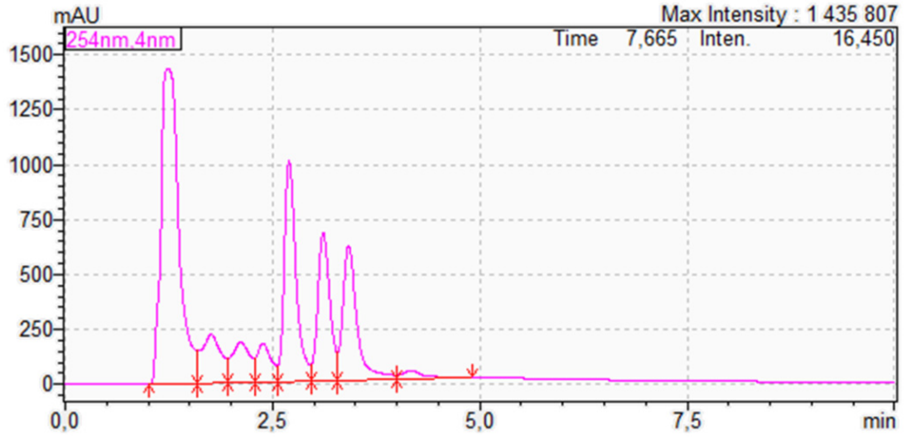

Peak	Compound	Ret. Time	Area	Area %	Height	Height %
1	unknown	1.238	21717258	39.401	1434068	32.997
2	unknown	1.760	3652129	6.626	221882	5.105
3	unknown	2.116	2706158	4.910	185161	4.260
4	unknown	2.384	2083826	3.781	175440	4.037
5	**4b**	2.699	9998134	18.139	1006105	23.150
6	**7b**	3.111	6941361	12.593	673831	15.504
7	**6b**	3.417	7280848	13.209	609976	14.035
8	unknown	4.162	739122	1.341	39583	0.911
Total			55118835	100.000	4346045	100.000

Figure S21. HPLC analysis of reaction mixture from reduction of **4b** in chloroform.

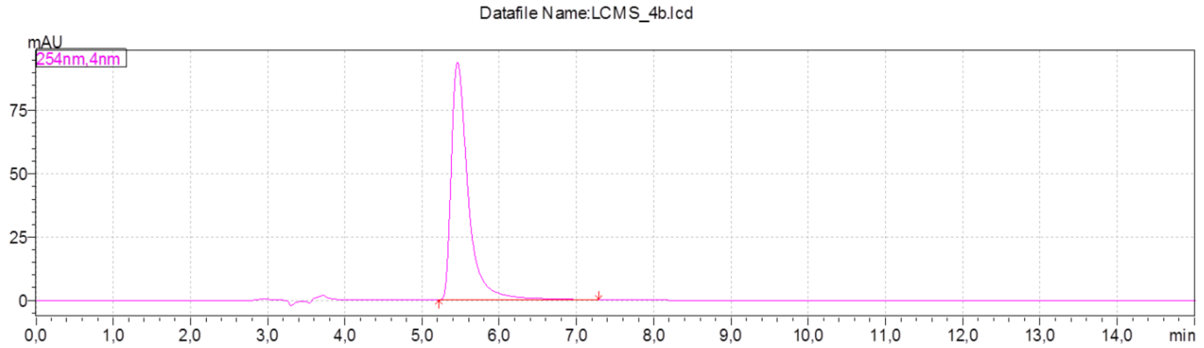

Peak#	Area%	Height%	Ret. Time	Area%	Height%
1	1421451	93725	5.458	100.000	100.000
Total	1421451	93725		100.000	100.000

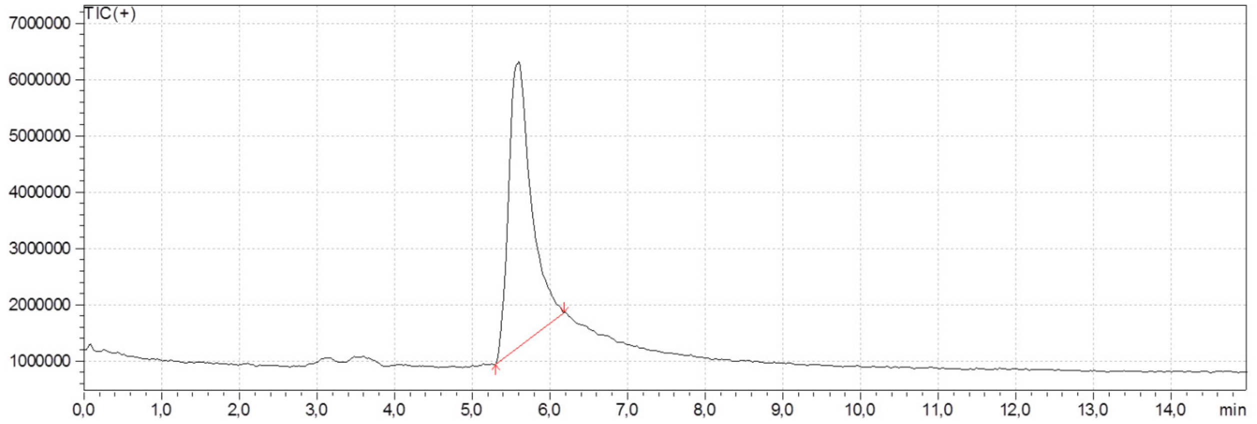

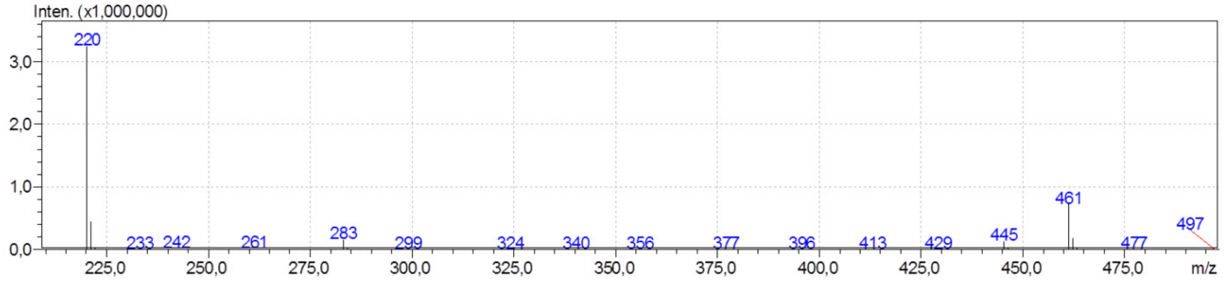

Raw Spectrum: [5.583 → 5.617]; Background: [5.300 → 6.183]; Base Peak: m/z 220.20

Peak#	m/z	Absolute Intensity	Relative Intensity
1	220.20	3252229	100.00
2	461.30	726357	22.33
3	221.20	448220	13.78
4	462.35	179505	5.52
5	283.15	165093	5.08
6	445.30	133355	4.10
7	222.20	38929	1.20
8	446.35	34725	1.07

Figure S22. LCMS data of **4b**.

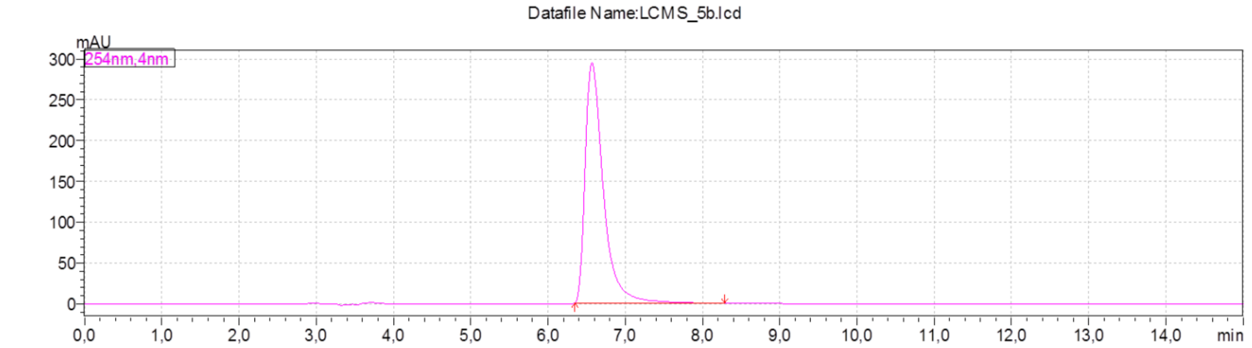

Peak#	Area%	Height%	Ret. Time	Area%	Height%
1	4811445	294582	6.569	100.000	100.000
Total	4811445	294582		100.000	100.000

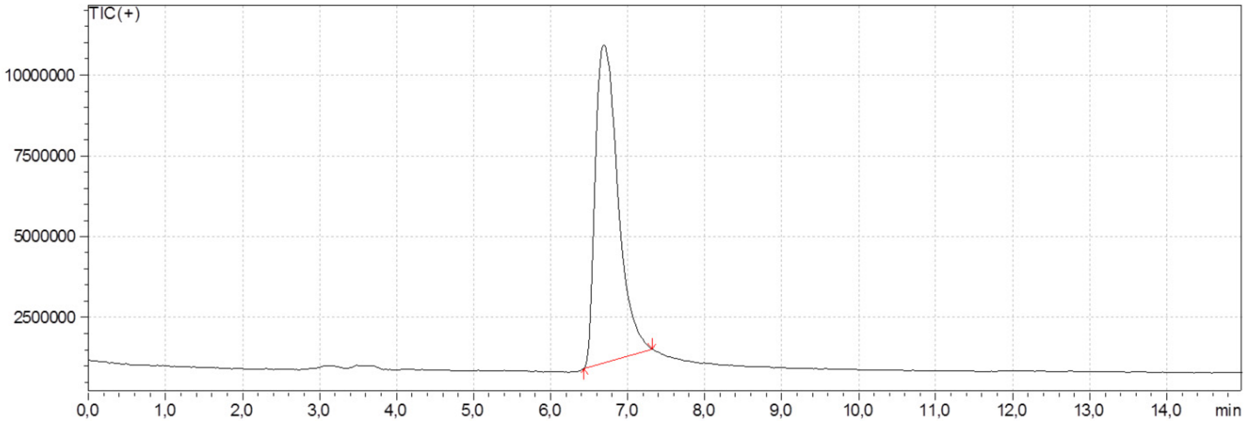

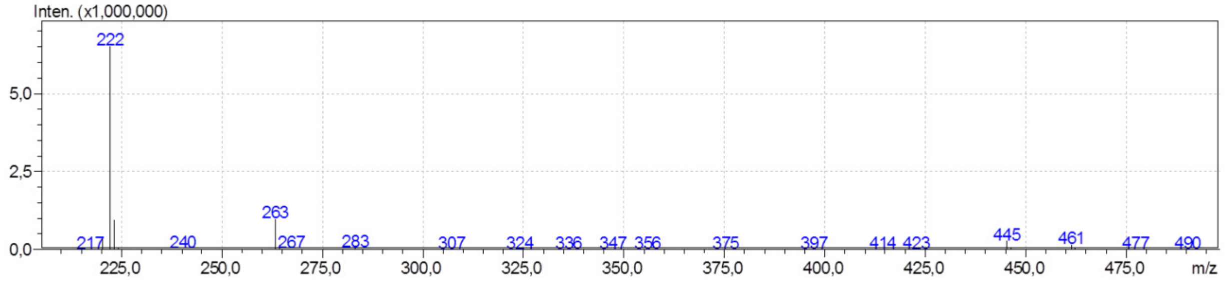

Raw Spectrum: [6.683 → 6.717]; Background: [6.433 → 7.317]; Base Peak: m/z 222.20

Peak#	m/z	Absolute Intensity	Relative Intensity
1	222.20	6531952	100.00
2	263.20	989592	15.15
3	223.25	968996	14.83
4	220.20	341758	5.23
5	445.30	282483	4.32
6	461.30	158200	2.42
7	264.25	154218	2.36
8	224.20	84418	1.29

Figure S23. LCMS data of **5b**.

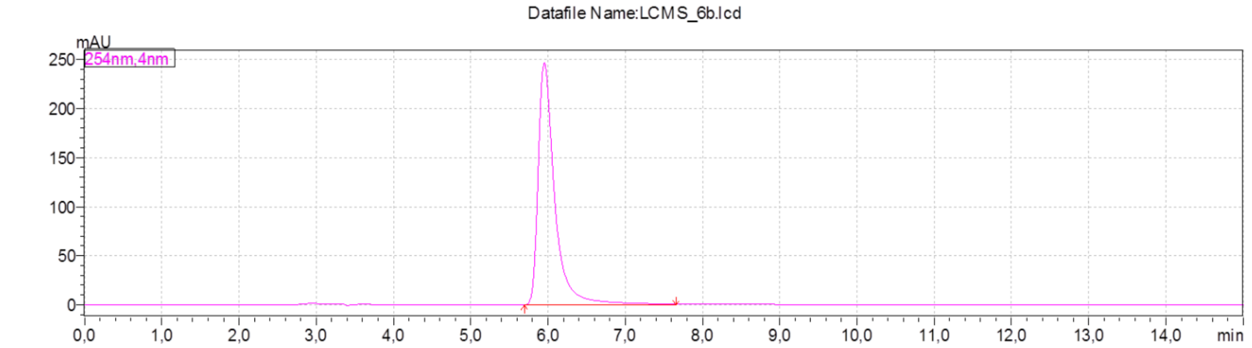

Peak#	Area%	Height%	Ret. Time	Area%	Height%
1	3702076	246882	5.953	100.000	100.000
Total	3702076	246882		100.000	100.000

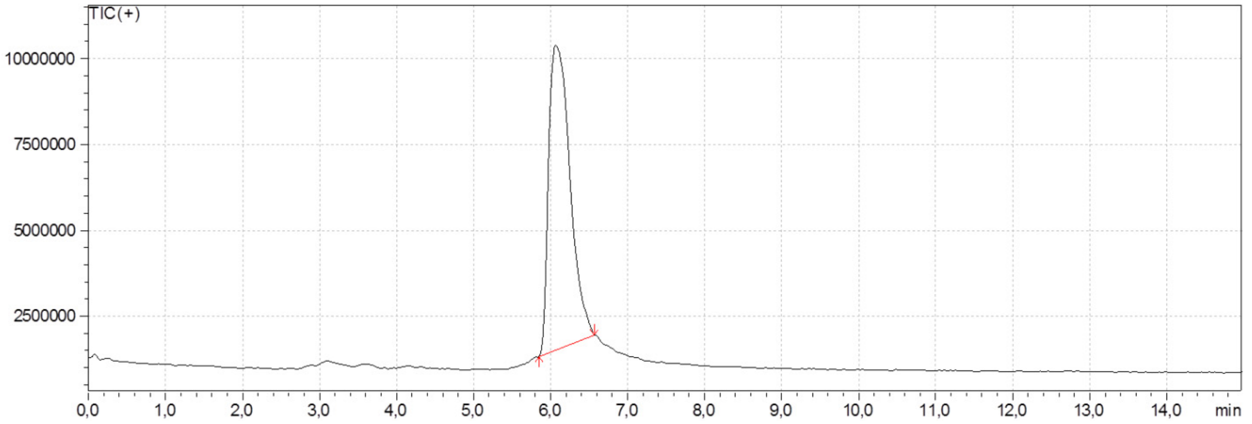

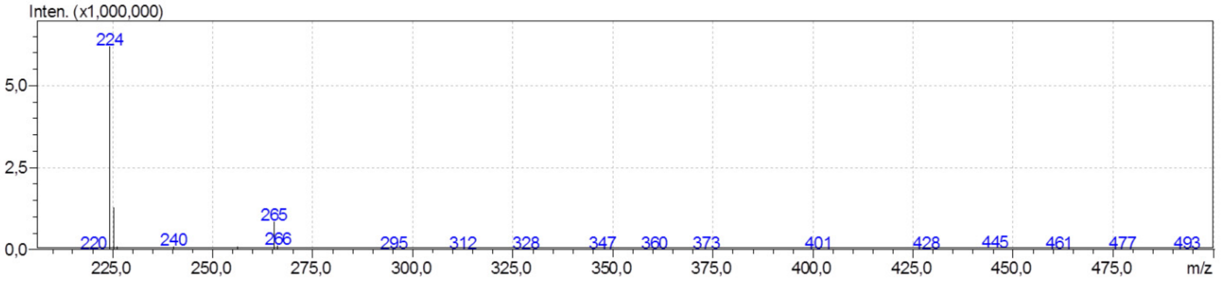

Raw Spectrum: [6.050 → 6.083]; Background: [5.850 → 6.567]; Base Peak: m/z 224.20

Peak#	m/z	Absolute Intensity	Relative Intensity
1	224.20	6217046	100.00
2	225.20	1294927	20.83
3	265.25	862018	13.87
4	266.25	134097	2.16
5	226.20	109506	1.76
6	240.20	109623	1.76
7	256.20	107292	1.73

Figure S24. LCMS data of **6b** (SYN).

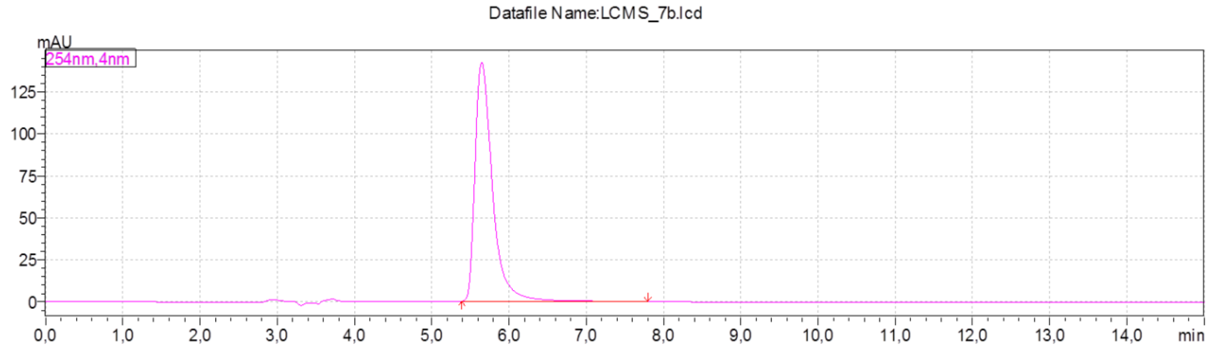

Peak#	Area%	Height%	Ret. Time	Area%	Height%
1	2522619	142061	5.647	100.000	100.000
Total	2522619	142061		100.000	100.000

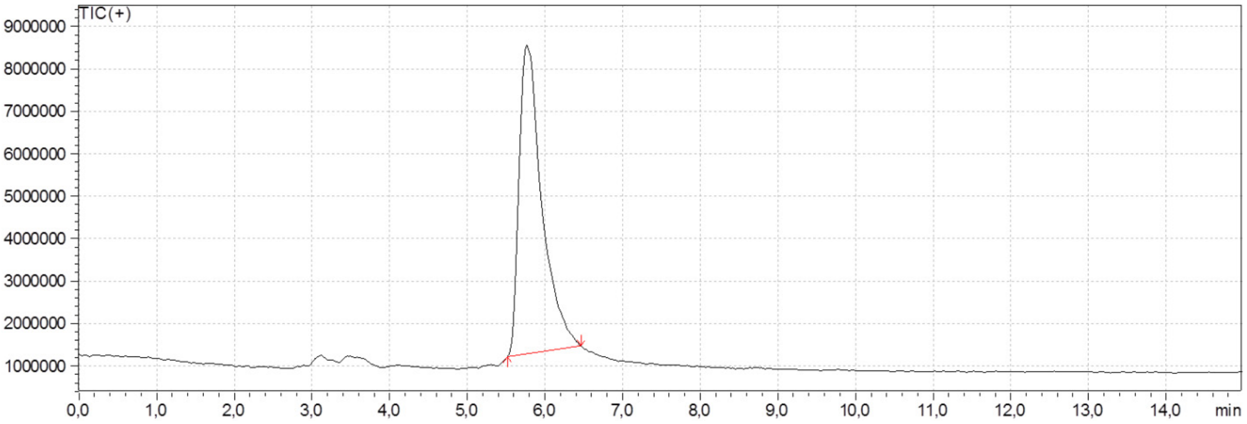

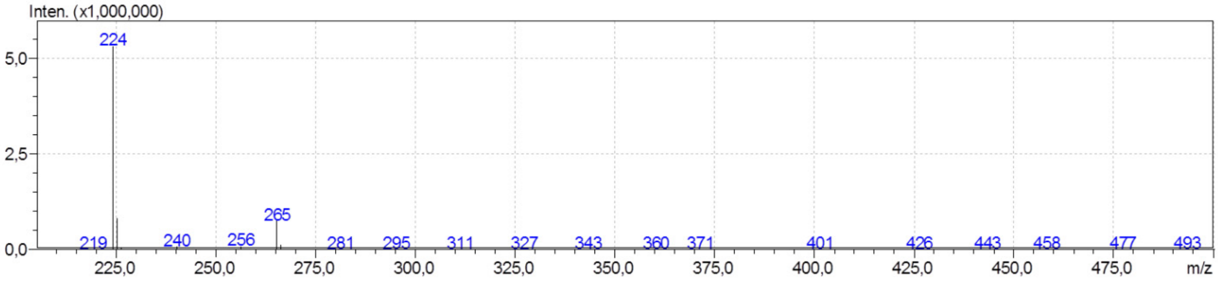

Raw Spectrum: [5.750 → 5.783]; Background: [5.517 → 6.467]; Base Peak: m/z 224.20

Peak#	m/z	Absolute Intensity	Relative Intensity
1	224.20	5333542	100.00
2	225.20	827126	15.51
3	265.25	753995	14.14
4	266.25	118885	2.23
5	256.20	86602	1.62
6	240.20	78722	1.48
7	226.20	71559	1.34

Figure S25. LCMS data of **7b (ANTI)**.
